# SNHG17 alters anaerobic glycolysis by resetting phosphorylation modification of PGK1 to foster pro-tumor macrophage formation in pancreatic ductal adenocarcinoma

**DOI:** 10.1186/s13046-023-02890-z

**Published:** 2023-12-15

**Authors:** Jiayu Lin, Yihao Liu, Pengyi Liu, Wenxin Qi, Jia Liu, Xingfeng He, Qian Liu, Zehua Liu, Jingxin Yin, Jiewei Lin, Haili Bao, Jianhong Lin

**Affiliations:** 1 Department of General Surgery, Ruijin Hospital, Shanghai Jiao Tong University School of Medicine, Shanghai, 200025 China; 2 School of Life Sciences, Shanghai University, Shanghai, China; 3 Department of Thoracic Surgery, Shanghai Pulmonary Hospital, Tongji University School of Medicine, Shanghai, China; 4 Department of Pharmacy, The Third Hospital of Xiamen, Xiamen, 361100 China; 5 Research Institute of Pancreatic Disease, Shanghai Jiaotong University School of Medicine, Shanghai, China; 6 Drug Research Program, Division of Pharmaceutical Chemistry and Technology, Faculty of Pharmacy, University of Helsinki, 00014 Helsinki, Finland

**Keywords:** TAMs, PDAC, SNHG17, Anaerobic glycolysis, miR-628-5p, PGK1

## Abstract

**Background:**

Within the tumor immune microenvironment (TME), tumor-associated macrophages (TAMs) are crucial in modulating polarization states to influence cancer development through metabolic reprogramming. While long non-coding RNAs (lncRNAs) have been shown to play a pivotal role in the progression of various cancers, the underlying mechanisms by which lncRNAs alter M2 polarization through macrophage metabolism remodeling remain unelucidated.

**Methods:**

RNA sequencing was used to screen for differentially expressed lncRNAs in TAMs and normal tissue-resident macrophages (NTRMs) isolated from pancreatic ductal adenocarcinoma (PDAC) tissues, whilst RT-qPCR and FISH were employed to detect the expression level of SNHG17. Moreover, a series of in vivo and in vitro experiments were conducted to assess the functions of SNHG17 from TAMs in the polarization and glycolysis of M2-like macrophages and in the proliferation and metastasis of pancreatic cancer cells (PCs). Furthermore, Western blotting, RNA pull-down, mass spectrometry, RIP, and dual-luciferase assays were utilized to explore the underlying mechanism through which SNHG17 induces pro-tumor macrophage formation.

**Results:**

SNHG17 was substantially enriched in TAMs and was positively correlated with a worse prognosis in PDAC. Meanwhile, functional assays determined that SNHG17 promoted the malignant progression of PCs by enhancing M2 macrophage polarization and anaerobic glycolysis. Mechanistically, SNHG17 could sponge miR-628-5p to release PGK1 mRNA and concurrently interact with the PGK1 protein, activating the pro-tumorigenic function of PGK1 by enhancing phosphorylation at the T168A site of PGK1 through ERK1/2 recruitment. Lastly, SNHG17 knockdown could reverse the polarization status of macrophages in PDAC.

**Conclusions:**

The present study illustrated the essential role of SNHG17 and its molecular mechanism in TAMs derived from PDAC, indicating that SNHG17 might be a viable target for PDAC immunotherapy.

**Supplementary Information:**

The online version contains supplementary material available at 10.1186/s13046-023-02890-z.

## Introduction

Pancreatic ductal adenocarcinoma (PDAC) is anticipated to become the second leading cause of cancer-related deaths by 2030 [1]. Although the application of standard treatments, such as surgical resection and chemotherapy, has improved survival rates, the 5-year survival rate remains a dismal 8% [2]. This is partly because the disease is frequently diagnosed at an advanced stage and has a high propensity for recurrence. While immunotherapy confers considerable benefits in solid organ tumors, it has failed to yield effective responses in PDAC cases [3, 4]. Thus, there is a pressing need to identify novel biomarkers and unfold underlying molecular mechanisms in order to enhance the therapeutic strategies for PDAC patients.

The poor prognosis of PDAC is associated with the tumor microenvironment (TME), in which a pronounced desmoplastic response generates a large stromal component consisting of immune cells, inflammatory cells, growth factors, extracellular matrix, and fibroblasts [5]. Indeed, the TME plays an instrumental role in the tumorigenesis and progression of PDAC [6]. Tumor-associated macrophages (TAMs) are a critical element of immune infiltration within tumor tissues [7].

A large proportion of TAMs are recruited and polarized from the peripheral blood by cytokines and chemokines secreted by tumors and stromal cells [8]. Macrophages can be categorized into two types: the classically activated M1-type and the alternatively activated M2-type. Within the TME, the majority of TAMs differentiate into the M2 phenotype, which promotes tumor progression, encompassing tumor growth, metastasis, and angiogenesis [9–11]. According to previous studies, a high degree of M2-TAM infiltration is correlated with a poor PDAC prognosis [12, 13]. Therefore, uncovering the regulatory mechanisms of TAM polarization could contribute to fine-tuning PDAC immunotherapy strategies.

Long non-coding RNAs (lncRNA) are non-coding RNAs exceeding 200 nucleotides long. A plethora of studies have highlighted the involvement of lncRNAs in numerous pathophysiological processes, including cell proliferation, invasion, and Epithelial-Mesenchymal Transition (EMT) [14–16]. Mechanistically, earlier research has demonstrated that lncRNAs may modulate tumor progression by binding to RNA-binding proteins, releasing mRNA as ceRNA to sponge cytoplasmic miRNAs, as well as regulating nuclear gene transcription [17–19]. Although several lncRNAs have been identified to play vital regulatory roles within the TME of PDAC [20, 21], their molecular functions in TAMs remain elusive. Hence, unraveling the molecular mechanisms of lncRNAs in TAMs is of paramount importance.

SNHG17 has emerged as an oncogene in several cancer types, including colorectal cancer, hepatocellular carcinoma, gastric cancer, and breast cancer, as it exhibits increased expression levels in these tumor types [22–25]. Its contribution to tumor progression is multifaceted, involving diverse mechanisms such as promoting cell proliferation, inhibiting apoptosis, fostering angiogenesis, and facilitating metastasis [22, 26]. Moreover, SNHG17 has been implicated in conferring resistance to chemotherapy and targeted therapies in specific cancers [27]. For instance, in gastric cancer, SNHG17 has been identified as an enhancer of resistance to the widely-used chemotherapy drug cisplatin [28]. It may achieve this by modulating the expression of genes implicated in drug metabolism, DNA repair, and apoptosis, thereby compromising the effectiveness of treatment [29]. While information regarding the role of SNHG17 in the tumor immune microenvironment remains limited, our study aims to delve into its involvement specifically in tumor-associated macrophages from PDAC.

In the current study, SNHG17 was highly expressed in PDAC-derived TAMs, validating that SNHG17 sponged miR-628-5p to enhance PGK1 expression. Additionally, SNHG17 did not only bind to the PGK1 protein but also recruited the ERK1/2 protein in the cytoplasm, thereby promoting phosphorylation modification of the PGK1 protein. This sequence of interactions enhanced the anaerobic glycolysis of TAMs and promoted alternative macrophage activation. These findings uncovered a novel perspective on the fine regulation of macrophage activation status, presenting a potential target for macrophage-based antitumor therapy.

## Materials and methods

### Clinical sample and magnetic-activated cell sorting (MACS)

Clinical samples were procured from PDAC patients who underwent pancreaticoduodenectomy at Ruijin Hospital, affiliated with Shanghai Jiaotong University Medical School, between July 2022 and March 2023. These patients were pathologically diagnosed with PDAC and did not receive neoadjuvant chemotherapy. After obtaining informed consent from each patient, a total of 30 pairs of pancreatic tumor tissues and their corresponding adjacent normal tissues were isolated during a surgical procedure and stored in Tissue Preservation Solution (Absin) on ice. For processing, the tissues were promptly dissected into small pieces in precooled RPMI-1640 and digested using the human Tumor Dissociation Kit (Miltenyi) at 37 °C for 20 min. Afterward, filtration through the Falcon® 40 µm Cell Screen (Corning) was completed, and single cells were prepped for magnetic sorting. PE anti-human CD163 Antibody (Biolegend) and PE anti-human CD11b Antibody (Biolegend) were employed for macrophages sorting, respectively. Thereafter, Anti-PE Microbeads (Miltenyi), LS Columns (Miltenyi), and MACS® MultiStand (Miltenyi) were used for positive selection. Details of the 30 PDAC patients are listed in Table S1.

### Cell culture, polarization, and co-culture system

PC lines (PANC-1 and PATU-8988), leukemic monocytes (THP-1), and Human embryonic kidney cells (HEK-293 T) were purchased from the Cell Bank of the Chinese Academy of Sciences (Shanghai, China) and authenticated through STR profiling. THP-1 and PATU-8988 cells were cultured in RPMI-1640 supplemented with 10% fetal bovine serum (FBS) (Gbico) and 1% Penicillin–Streptomycin Solution (P/S) (NCM), while PANC-1 and HEK-293 T cells were cultured in DMEM supplemented with 10% FBS and 1% P/S. All cells were cultured at 37 °C in an atmosphere containing 5% CO_2_. Additionally, to induce the polarization of THP-1 cells to M2 macrophages, the cells were incubated in 100 ng/mL phorbol-12-myristate-13-acetate (PMA) for 48 h, followed by incubation in 20 ng/mL IL-4 for 48 h. For co-culturing purposes, IL-4 was replaced by the supernatant of PCs to induce the polarization of THP-1 cells. For primary macrophages from mice, BMDM cells were isolated from bone marrow of C57BL-6 mice similar to previously reported [30]. BMDM and Pan02 cells were co-cultured in DMEM supplemented with 10% fetal bovine serum (FBS) (Gbico) and 1% Penicillin–Streptomycin Solution (P/S) (NCM). For primary macrophages from healthy donor patients, monocytes were isolated from peripheral blood by MACS (Magnetic Activated Cell Sorting). Monocytes were treated with 50 ng/mL M-CSF (ThermoFish, PHC9504) for 6 days and co-cultured with PANC-1 cells for 2 days.

### Organoid culture and measure

Pancreatic cancer organoids were extracted from the tumor tissues of PDAC patients who underwent surgery at Ruijin Hospital. PDAC tissues were digested by the human Tumor Dissociation Kit (Miltenyi), as mentioned above. After filtration, the cells were seeded into Matrigel (Corning), wherein they were subsequently cultured in a complete organoid culture medium, OmaStem® Pan-cancer Advanced (OmaStem). Organoids were then digested by TrypLETM Express (ThermoFisher), seeded in 96-well plates, and imaged every 5 days. The relative activity of the organoids was measured using the CellTiter-Glo® 3D Cell Viability Assay (Promega) according to the manufacturer’s instructions.

### Plasmids and transfection

For transient transfection, appropriate concentrations of plasmids were introduced into the cell supernatant after gently mixing with Hilymax (Dojindo). After an 8-h interval, the culture medium was changed. mRNA and protein expression levels were assessed three days later. For stable transfections, the appropriate lentivirus was added to the supernatant, and the medium was regularly changed. Following expression verification, cells were treated with 2 μg/mL puromycin to identify cells expressing the resistance gene, which represented stably transfected cell lines. All plasmids and lentiviruses were sourced from Bioegene (Shanghai, China). For this study, the following were transfected into THP-1 cells: negative control (NC), short-hairpin RNA (shRNA), overexpressing and mutant sequences of SNHG17; miR-628-5p mimics and inhibitor; NC, shRNA and mutants of PGK1; NC, shRNA, and overexpression of ERK1/2. The sequences of plasmids and lentiviruses are summarized in Table S2.

### RNA extraction and quantitative real-time PCR (qRT-PCR)

RNA was extracted from cells using the SteadyPure Universal RNA Extraction Kit (Accurate Biology) and then reverse transcribed to cDNA using the Evo M-MLV reverse transcription kit (Accurate Biology). Post-transcription, the concentration and purity of the RNA were determined. Relative RNA expression levels were detected using the Evo M-MLV One-Step RT-qPCR Kit (SYBR) on qTOWER384G (Analytik Jena). Primer sequences for this analysis are delineated in the Table S3.

### Western blot and Co-immunoprecipitation (Co-IP)

Proteins were lysed from cells by RIPA buffer (ABclonal) with 1% Protease inhibitor cocktail (MCN Biotech) on ice for 10 min. The Pierce BCA assay kit (ThermoFisher) was used for protein quantification. A loading buffer was then added to the proteins, which was heated for denaturation. Regarding the Western blot procedure, the proteins were subjected to SDS-PAGE separation and transferred onto PVDF membranes. Following blocking by Protein Free Rapid Blocking Buffer (Epizyme), the membranes were incubated in the appropriate primary and secondary antibodies to determine the expression of specific proteins. As for the Co-IP process, Protein A/G Magnetic Beads (ThermoFisher) were employed to incubate the proteins and specific antibodies or control IgG on rotation at 4 °C overnight. The selected samples were then used for immunoblotting. Details of antibodies are listed in Table S4.

### Flow cytometry (FC)

Cells were harvested, centrifuged, washed, and suspended in 100 μL pre-cooled 1% BSA solution (dissolved in PBS (CR0014-500ML(Shandong Sparkjade Biotechnology Co., Ltd.))). They were then stained with flow antibodies conjugated with the indicated fluorescence for half an hour by following the recommended concentration in the dark. CytoFLEX S (Beckman) recorded corresponding fluorescence signals after unbound antibodies were discarded. Various groups, including a blank group, a single antibody-stained group, and a sample group, were used for voltage adjustment and compensation. A detailed list of the antibodies is available in Table S4.

### Cell proliferation assay

Cell proliferation was measured with Cell Counting Kit-8 (CCK-8) (Meilune Bio). Briefly, the cells were seeded in 96-well plates at a density of 1000 cells/well and incubated with DMEM enriched with 10% CCK-8 for 2 h. Absorbance was measured at 450 nm every 24 h for 5 consecutive days. For the colony formation assay, the cells were seeded in 6-well plates at a density of 1000 cells/well and incubated for approximately 2 weeks. The resulting colonies were stained with 1% crystal violet and subsequently imaged.

### Transwell assay

The migratory and invasive abilities of the cells were determined via Transwell assay (Corning). Briefly, 100,000 PCs were seeded in the upper chamber suspended in 200 μL FBS-free medium, with 700 μL conditional medium in the lower chamber. Migrated cells were stained with 1% crystal violet and pictured under a microscope after 48 h. For the invasion assay, the chamber membranes were coated with Matrigel (Corning), and the remaining steps were identical to the migration assay.

### Immunohistochemistry (IHC), Immunofluorescence (IF), and RNA Fluorescence In Situ Hybridization (FISH) assays

Tissues were formalin-fixed, paraffin-embedded, and sectioned onto slides. IHC staining was thereupon performed using the standard streptavidin–biotin-peroxidase complex method. Following deparaffinization and rehydration, the slides were successively subjected to antigen retrieval, inactivation, incubation with primary and secondary antibodies, DAB staining, and sealing. Finally, representative pictures were captured under a microscope, and IHC scores were assessed based on the area and degree of staining. The procedures for IF and FISH were similar to those for IHC, except for the staining process; pictures were also taken through confocal microscopy (Zeiss). FISH probes were purchased from Bioegene (Shanghai, China). The sequence of FISH probes of SNHG17 is 5’-GCTCTGGTGACGCTTCATGTGGTAGCCTCACTCTC-3’. The cells were fixed in 4% formalin, and the cell membranes were ruptured using Triton X-100. Nuclei were stained with DAPI (Beyotime) to allow for visualization and analysis of lncRNA subcellular localization.

### Dual‑Luciferase reporter assay

Plasmids were purchased from Bioegene (Shanghai, China). HEK-293 T cells were cultured in 6-well plates and used for transient transfection. SNHG17, miR-628-5p, PGK1, and their matched 3’-UTR mutation sequences were transfected into HEK-293 T cells. Luciferase activity was analyzed using the Dual-Luciferase Reporter Assay Kit (Vazyme) 48 h after transfection, following the manufacturer’s instructions.

### Seahorse assay

THP-1 cells were seeded into Seahorse XFe96 FluxPak plates (Agilent) at a density of 15,000 cells per well to differentiate into M2-like macrophages. For the glycolytic stress test, 10 mmol/L glucose, 2 μmol/L oligomycin, and 50 mmol/L 2-deoxyglucose (2-DG) were sequentially added to each well. As for the mitochondrial stress test, 1 μmol/L oligomycin, 2 μmol/L FCCP, 0.5 μmol/L rotenone, and 0.5 mmol/L actinomycin A were added to the wells, respectively. The oxygen consumption rate (OCR) and extracellular acidification rate (ECAR) of each well were automatically determined by the Agilent Seahorse XFe96 analyzer and processed with the Seahorse Wave Desktop software.

### Glucose and lactate acid measurements

THP-1 cells were induced to differentiate into M2-like macrophages in 24-well plates. Glucose uptake capacity was measured using the Glucose Uptake Test Kit—Green (Dojindo) according to the manufacturer’s instructions. The fluorescent glucose analog probe was competitively bound to glucose transport receptors, and fluorescence intensity was detected through fluorescence microscopy and flow cytometry. Lactate acid in the supernatant was measured using the L-lactic acid (L-LA) content test kit (Solarbio) following the manufacturer’s instructions. Absorbance was measured at 570 nm to calculate L-LA levels based on the standard curve.

### RNA Immunoprecipitation (RIP)

The Magna RIP™ RNA-Binding Protein Immunoprecipitation Kit (Millipore) was employed to perform the RIP assay, according to the manufacturer’s instructions. The concentration and quality of RNAs were tested prior to experimentation. Enriched RNAs were subsequently used for qRT-PCR, as previously described. Details of antibodies are presented in Table S4.

### RNA pull-down assay

The Pierce™ Magnetic RNA–Protein Pull-Down Kit (Pierce) was used to detect the proteins binding to lncRNA. Biotinylated RNA was transcribed in vitro, incubated with cell lysates at 4 °C, and captured using streptavidin–agarose beads (Invitrogen). Finally, the RNA-binding proteins were identified by mass spectrometry and Western blotting.

### Mouse models

BALB/c nude mice were selected for in vivo studies. THP-1 cells or and pancreatic cancer cells were co-injected into the spleen of nude mice. BMDM cells which were knocked down SNHG17 and Pan02 cells were co-injected into the spleen of C57BL/6 mice. Six-week-old male nude mice were randomly assigned to 4 groups. For the subcutaneous tumors, roughly 4 × 10^6^ cells in 150 μL PBS were injected into the shoulder region of the mice, with a THP-1 cell or BMDM cell to PC ratio of approximately 1:3. Three to four weeks later, the mice were euthanized, and the subcutaneous tumors were collected, photographed, weighed, and stained with hematoxylin and eosin (HE) for IHC analysis. Overall survival curves were plotted as well. In order to investigate metastatic capacity in vivo, mice were anesthetized, and about 1 × 10^6^ cells were suspended in 50 μL PBS and injected into the spleen. Similarly, the livers were stained and imaged for analysis.

### Statistical analysis

Each experiment was performed in triplicate. The Student’s two-tailed unpaired t-test, one-way ANOVA, Mann–Whitney, Kruskal–Wallis tests and Chi-square test were used for statistical analyses. The Kaplan–Meier (K-M) method was adopted for survival curves. Data were calculated using GraphPad Prism Version 9.0 and presented as mean ± standard deviation (SD). A *p*-value of less than 0.05 (*p* < 0.05) was considered statistically significant.

## Results

### High expression of SNHG17 in TAMs had clinical significance in PDAC

**Fig. 1:**
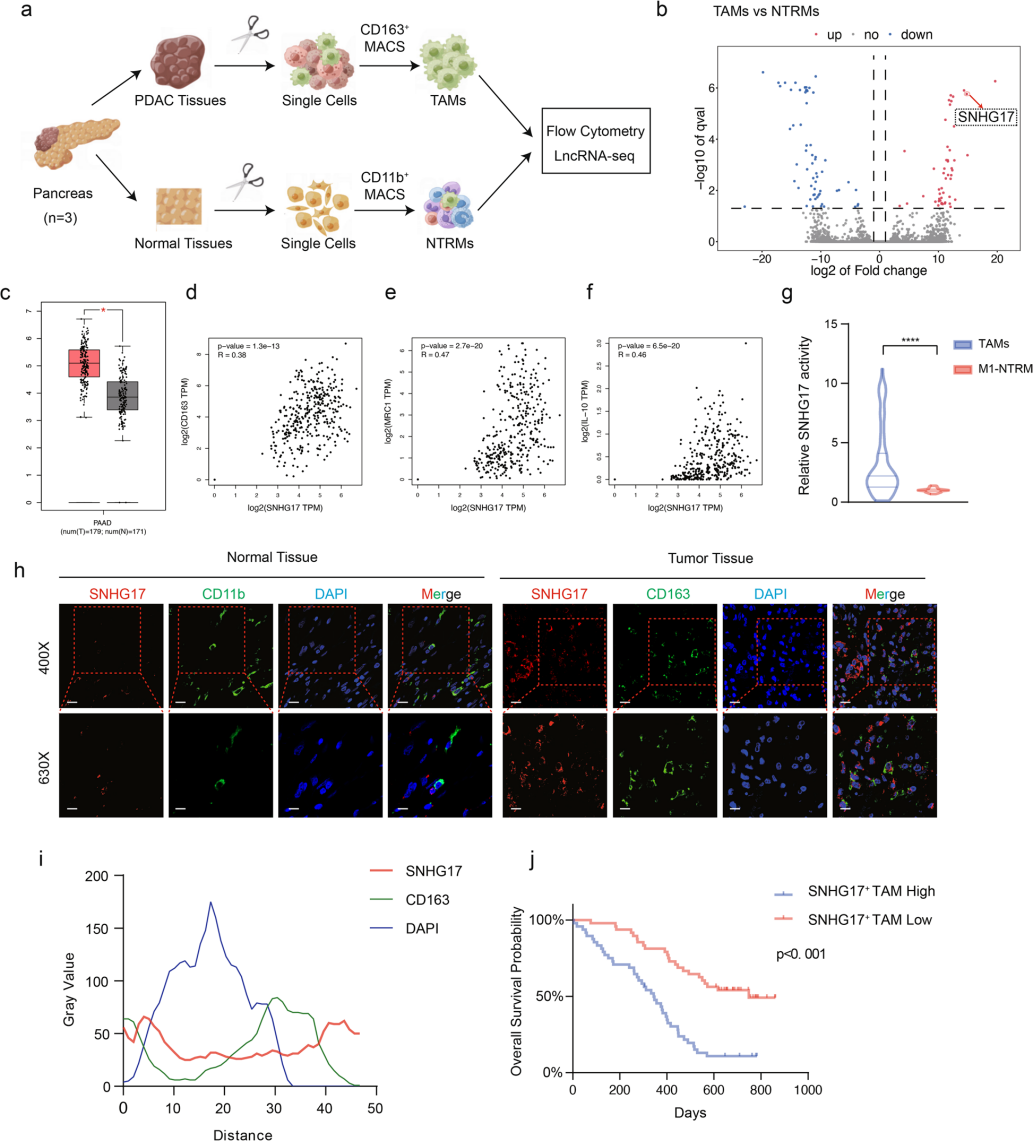
SNHG17 was overexpressed in TAMs from PDAC and associated with a poor prognosis in PDAC. **A** Schematic representation of the experimental workflow, including MACS (Magnet-Activated Cell Sorting) of TAMs and NTRMs from three PDAC patients and lncRNA sequencing of TAMs and NTRMs. **B** Volcano plot illustrating the differential expression level of lncRNAs between TAMs and NTRMs. **C**. The expression level of SNHG17 in tumor tissues (*n* = 179) and healthy tissues (*n* = 171) from the TCGA dataset. **D**-**F** Spearman correlation between SNHG17 and CD163 (D) or MRC1 (**E**) or IL10 (**F**) in PAAD from TCGA dataset. **G** The expression of SNHG17 in TAMs and NTRMs sorted from 30 PDAC patients. **H** Colocalization of SNHG17 (red) and CD163 or CD11b (green) in 96 clinical samples of PDAC as determined by fluorescence microscopy. DAPI staining (blue) displaying nuclei (DNA). Scar bar: up: 20 μm, down: 12 μm. **I** Intensity of immunofluorescence staining for SNHG17, CD163, and DAPI in tissue microarrays from 96 PDAC patients. Red represents SNHG17. Green represents CD163. Blue represents DAPI. **J** K-M survival curve of PDAC patients in the SNHG17 high expression and low expression groups in TAMs. *P < 0.05; **P < 0.01; ***P < 0.001; ****P < 0.0001

Previous studies have identified that TAMs play a key immunosuppressive role in PDAC [31]. In order to explore functional lncRNAs during the development of pro-carcinogenic macrophages, CD163^+^ cells and CD11b^+^ cells were isolated from tumor tissues and paracancerous tissues of three PDAC patients and labeled as TAMs and NTRMs, respectively. LncRNA sequencing was subsequently performed (Fig. 1a). A total of 111 differentially expressed lncRNAs (50 up-regulated and 61 down-regulated) could be identified, with a significant elevation in the expression of SNHG17 in TAMs compared to NTRMs (Fig. 1b). Next, the expression level of SNHG17 in pancreatic cancer was further analyzed using GEPIA2. The results revealed that SNHG17 was highly expressed in tumor tissues and positively correlated with the level of TAM markers CD163, CD206, and IL-10, implying that SNHG17 not only played an essential role in the malignant progression of PDAC but was also closely associated with TAMs. (Fig. 1c-f). Thereafter, a FISH assay was carried out on tissue microarrays (TMAs) composed of paired tumor tissues and paracancerous tissues from 96 PDAC patients (Table S5). At the same time, TAMs and NTRMs were extracted from 30 paired tumor tissues and non-tumor tissues at Ruijin Hospital for RT-qPCR (Table S1). The expression level of SNHG17 was higher in TAMs compared to NTRMs, and SNHG17 had a strong degree of co-localization with CD163 in tumor tissues (Fig. 1g-i). To further explore the prognostic value of SNHG17-positive TAMs in pancreatic cancer, the mean fluorescence intensity of SNHG17 was evaluated in TMAs, and 96 PDAC patients were stratified into high- and low-expression SNHG17 groups based on the median value of mean fluorescence intensity. According to the K-M-plot, higher infiltration of SNHG17-positive macrophages correlated with worse clinical outcomes in PDAC (Fig. 1j). The aforementioned results signaled that SNHG17 was highly expressed in M2-like macrophages and contributed to M2-like macrophages driving the malignant progression of PDAC.

### SNHG17 promoted the proliferation and metastasis of PCs by facilitating the polarization of M2-like Macrophages In Vivo and In Vitro

**Fig. 2:**
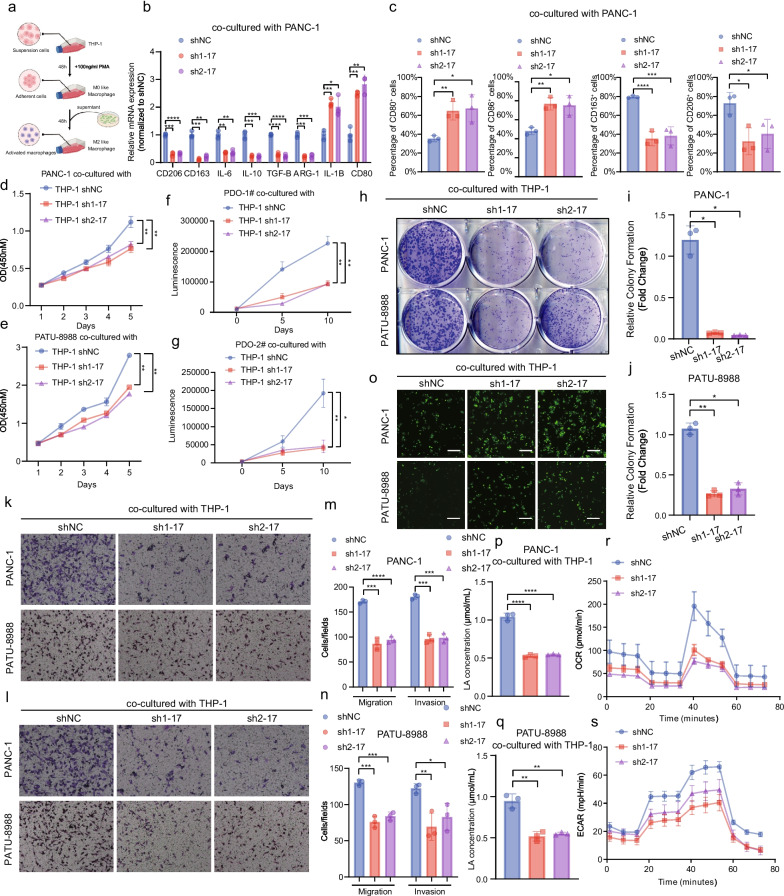
SNHG17 in THP-1 cell-derived TAMs enhanced anaerobic glycolysis and promoted M2 polarization and malignant progression of PCs. **A** Schematic representation of induction of THP-1 into TAMs in vitro. **B** The mRNA expression of M2 markers (CD206, CD163, IL6, IL10, TGFB and Arg1) and M1 markers (IL1B and CD80) after knockdown of SNHG17 in THP-1 derived TAMs. **C** Flow Cytometry ananlysis of CD80/CD86/CD163/CD206 expression in THP-1 derived TAMs (shNC, sh1-SNHG17 and sh2-SNHG17). **D**-**E** CCK8 analysis of PANC1 co-cultured with THP-1 derived TAMs (**D**) and PATU-8988 co-cultured with THP-1 derived TAMs (**E**). **F**-**G** CTG analysis of two patient-derived organoids (PDO1# and PDO2#). (**H**) Representative colony formation images of PANC1 co-cultured with THP-1 derived TAMs (shNC, sh1-SNHG17 and sh2-SNHG17) and PATU-8988 co-cultured with THP-1 derived TAMs (shNC, sh1-SNHG17 and sh2-SNHG17). **I**-**J** Colony numbers of PANC-1 and PATU-8988 in each group. **K**-**L** Representative migration (**K**) and invasion (**L**) images of PANC1 co-cultured with THP-1 derived TAMs (shNC, sh1-SNHG17 and sh2-SNHG17) and PATU-8988 co-cultured with THP-1 derived TAMs (shNC, sh1-SNHG17 and sh2-SNHG17). **M**–**N** Migrated and invaded PANC-1cells (**M**) per field and PATU-8988 cells (**N**) per field in each group (shNC, sh1-SNHG17 and sh2-SNHG17). **O**-**Q** Glucose uptaking (**O**) and Lactic acid concentration (**P**, **Q**) analysis of THP-1 derived TAMs in each group (shNC, sh1-SNHG17 and sh2-SNHG17). **R**-**S** Seahorse analysis of THP-1 cells (shNC, sh1-SNHG17 and sh2-SNHG17) co-cultured with PANC-1 cells. *P < 0.05; **P < 0.01; ***P < 0.001; ****P < 0.0001

To comprehend the biological function of SNHG17 in TAMs, a TAM model wherein THP-1 cells were co-cultured with either PANC-1 or PATU-8988 cells in vitro was constructed, as detailed in previous studies (Fig. 2a) [13]. Then, the expression of M1 and M2 markers after the knockdown of SNHG17 in THP-1 cells-derived TAMs was examined. According to RT-qPCR analysis, the expression of the pro-tumor macrophage markers CD206, CD163, IL-6, IL-10, Arginase-1, and TGF-β were downregulated compared to the normal control group (Fig. 2b and Figure S1a). However, the expression levels of M1-like macrophage markers CD80 and IL-1β were elevated. Moreover, these findings were in line with those of flow cytometry (Fig. 2c and Figure S1b-e). To better characterize the altered pattern of cytokines in the absence of SNHG17, we detected the levels of main cytokines related to TAMs (IL-6, IL-10 and TGF-β) and found that these cytokines were downregulated in SNHG17-interfered THP-1 derived TAMs (Figure S1f-g). After SNHG17 was knocked down in THP-1 cells-derived TAMs, the proportion of CD206^+^ and CD163^+^ TAMs decreased. These findings inferred that SNHG17 could promote the polarization of M2-like macrophages. An earlier study documented that M2-like macrophages could boost the proliferation and metastasis of surrounding PCs and promote the malignant progression of tumors by secreting cytokines such as IL-6 and IL-10 [32]. Therefore, the pro-tumorigenic functions of SNHG17 in M2-like macrophages were further investigated using co-culture models. Of note, the results of the colony formation, organoid proliferation and CCK-8 assays demonstrated that SNHG17 enhanced the proliferative activity of PCs (Fig. 2d-j and Figure S2a-d). Subsequently, the Transwell assay determined that PATU-8988 or PANC1 cells co-cultured with THP-1 cells with downregulated SNHG17 expression displayed weaker migratory and invasive abilities (Fig. 2k-n). Furthermore, we found that SNHG17 was able to alter the glycolysis level of THP-1 cell- derived TAMs (Fig. 2o-s and Figure S2e, f).

Additionally, to enhance the robustness of the function of SNHG17, we obtained the primary macrophages from mice and healthy donor patients and knocked down SNHG17. And we established two TAM models that BMDM were co-cultured with Pan02 cells to get BMDM-derived TAMs (BDT) and monocytes were treated with M-CSF and co-cultured with PANC-1 cells to get Monocyte-derived TAMs (MDT) (Figure S3a). The levels of M2 polarization were reduced after knockdown of SNHG17 in BDT and MDT (Figure S3b-k). Then, we found that the proliferation and metastasis of Pan02 co-cultured with BDT and PANC-1 co-cultured with MDT were down-regulated after knockdown of SNHG17 in BDT or PANC-1 (Figure S4a-i). Similarly, we identified that SNHG17 was shown to alter glycolysis levels in BDT and MDT (Figure S4j-l).

**Fig. 3:**
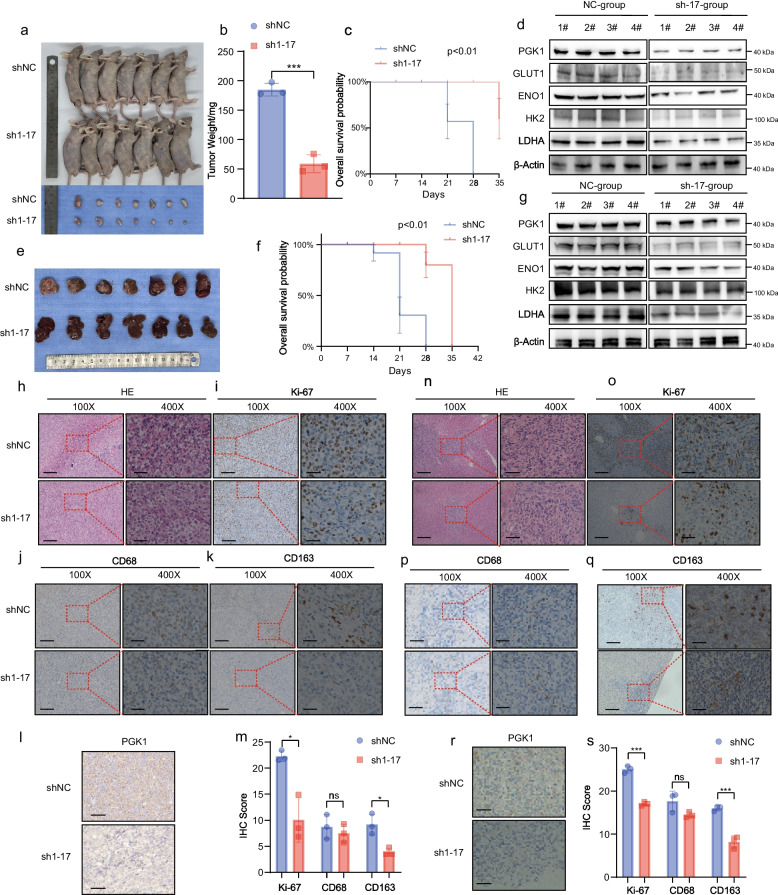
SNHG17 in THP-1 cell-derived TAMs boosted growth and metastasis of PCs in vivo. **A** Images of BALB/c nude mice which were injected with THP-1 cells (shNC and sh1-SNHG17) and PANC-1 cells. **B** Weights of subcutaneous tumors from BALB/c nude mice in each group. (*n* = 6) (**C**) and (**F**) K-M survival curves for BALB/c nude mice from the subcutaneous tumors mouse model (**C**) and liver metastasis mouse model (**F**) in the shNC and sh1-SNHG17 groups. **D** Images of livers from BALB/c nude mice co-injected with THP-1 cells and PANC-1 cells in the spleen. **D** and **G** Protein expression levels (PGK1, GLUT1, ENO1, HK2, and LDHA) in macrophages isolated from subcutaneous tumors (**D**) and metastatic lesions (**G**). (*n* = 4) (**H**) and (**N**) HE stained images of tissues from subcutaneous tumors mouse model (**H**) and liver metastasis mouse model (**N**) left: scale bar = 400 μm; right: scale bar = 100 μm. (**I**-**L**) and (**O**-**R**) IHC staining images of Ki-67 (**I**, **O**), CD68 (**J**, **P**), CD163 (**K**, **Q**), and PGK1(**L**, **R**) in tissues from the subcutaneous tumors mouse model and liver metastasis mouse model. left: scale bar = 400 μm; right: scale bar = 100 μm. (**M**) and (**S**) IHC score (Ki-67, CD68, CD163, and PGK1) of tissues from the subcutaneous tumors mouse model (**M**) and liver metastasis mouse model (**S**) in the shNC and sh1-SNHG17 groups. *P < 0.05; **P < 0.01; ***P < 0.001; ****P < 0.0001

Next, the effects of SNHG17 on tumor growth and metastasis were further validated in vivo using mouse models. THP-1 cells with SNHG17-knockdown were added to PANC-1 cells in a 1:3 ratio and thereupon injected into the subcutaneous layer or spleen of nude mice (*n* = 7) (Fig. 3a-e). BDT cells with SNHG17-knockdown and Pan02 cells were co-injected into subcutaneous layer or spleen of C57BL/6 mice (Figure S5a-d). As anticipated, the weight of the subcutaneous tumors of the mice in the SNHG17 sh1 group were considerably lower (Fig. 3b and Figure S5a-b); longer overall survival was also recorded for these mice, implying a better prognosis (Fig. 3c). Similar results were observed in the liver metastasis model (Fig. 3e, f and Figure S5c-d). In addition, as portrayed in Fig. 3d and g, the expression of multiple glycolytic genes was remarkably downregulated in the SNHG17 sh1 group in both subcutaneous and metastatic tumors. The subcutaneous tumor tissues were also subjected to IHC staining. The findings revealed that the SNHG17 sh1 group had a lower level of CD163^+^ macrophage infiltration and fewer Ki-67-positive cells compared with the shNC group, demonstrating that SNHG17 knockdown could reduce the polarization of pro-tumor macrophages and suppress tumor proliferation in vivo (Fig. 3h-m). The SNHG17 sh1 group presented with considerably fewer liver metastatic cells and a better prognosis than the shNC group (Fig. 3n). Finally, IHC staining portrayed that mice in the SNHG17 sh1 group had fewer CD163^+^ cells and lower proliferative activity in the liver (Fig. 3o-s).

In short, SNHG17 could induce the polarization of pro-tumor macrophages, facilitating the growth and metastasis of PCs.

### SNHG17 enhanced aerobic glycolysis in TAMs by sponging miR-628-5p to increase PGK1 mRNA expression

**Fig. 4:**
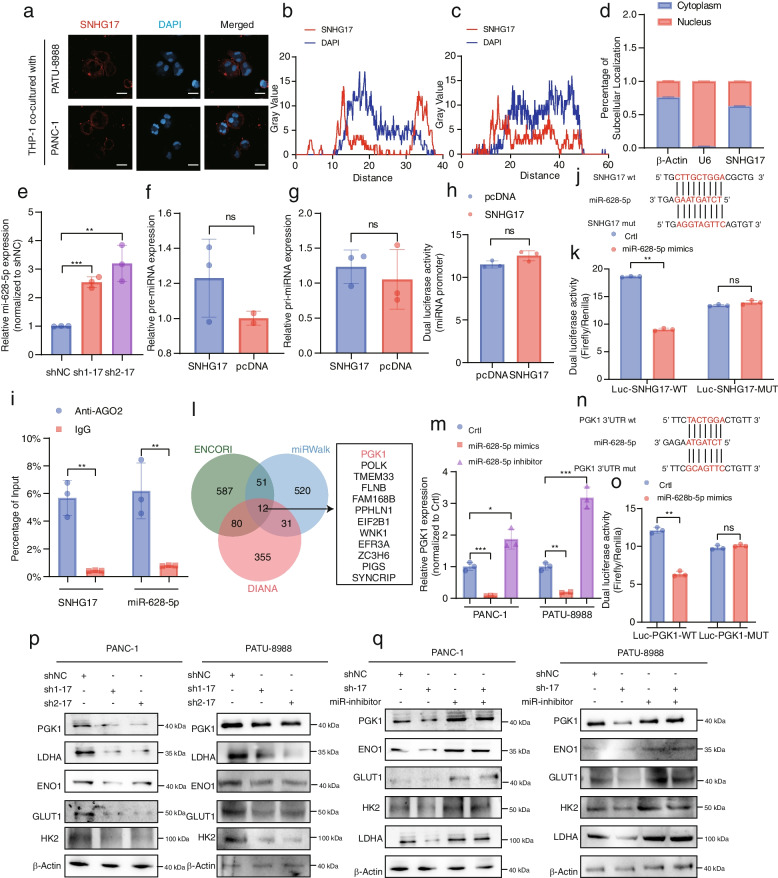
SNHG17 sponged miR-628-5p to release PGK1 mRNA. **A** FISH visualization of SNHG17 (red) in THP-1 cells following co-culture with either PATU-8988 or PANC-1 cells. Nucleus was stained with DAPI (blue). Scale bar: 20 μm. **B**-**C** Representative FISH images were analyzed to determine the intensity values for SNHG17 and DAPI in THP-1 cells-derived TAMs. Red represents SNHG17. Blue represents DAPI. **D** Relative expression levels of SNHG17 in the cytoplasm and nucleus of THP-1 cell-derived TAMs. **E** The RNA expression level of miR-628-5p in THP-1 cell-derived TAMs (shNC, sh1-SNHG17, and sh2-SNHG17). **F**-**G** Expression level of pre-miR-628-5p (**F**) and pri-miR-628-5p (**G**) in THP-1 cell-derived TAMs transfected with negative control plasmids (pcDNA) or SNHG17 plasmids. **H** Dual-luciferase assay was used to assess the ability of miR-628-5p to bind to the SNHG17 promoter region. **I** RIP analysis of THP-1 cell-derived TAMs indicated that AGO2 could interact with SNHG17 and miR-628-5p. **J**-**K** Dual-luciferase activity in HEK-293 T cells post-cotransfection with SNHG17 (WT or MUT) and miR-628-5p mimics. **L** Schematic representation of the screening process for downstream target genes of miR-628-5p. **M** The expression level of SNHG17 in THP-1 cell-derived TAMs transfected with miR-628-5p inhibitor or miR-628-5p mimics. **N**–**O** Dual-luciferase activity in HEK-293 T cells post-cotransfection with PGK1 (WT or MUT) and miR-628-5p mimics. **P** The protein expression level of PGK1, GLUT1, ENO1, HK2, and LDHA in THP-1 cell-derived TAMs (co-cultured with either PANC-1 cells or PATU-8988 cells) transfected with negative control, sh1-SNHG17, or sh2-SNHG17 plasmids. **Q** WB assays indicated that miR-628-5p could reverse the SNHG17-induced promotion of aerobic glycolysis in THP-1 cell-derived TAMs. *P < 0.05; **P < 0.01; ***P < 0.001; ****P < 0.0001

The subcellular location of lncRNA largely dictates its function [33]. FISH and subcellular isolation assay highlighted that SNHG17 was primarily localized in the cytoplasm of TAMs (Fig. 4a-d). Existing studies have posited that cytoplasmic lncRNAs act as ceRNAs to sponge miRNAs; hence, the hypothesis that SNHG17 might competitively bind with miRNAs in the cytoplasm was proposed [34]. The potential targets of SNHG17 were predicted using the miRTarBase website and then validated by qPCR. miR-628-5p exhibited a dramatic upregulation upon SNHG17 knockdown in TAMs (Fig. 4e). Then, miR-628-5p expression was detected in 30 matched pairs of clinical specimens. As depicted in Figures S6a and b, miR-628-5p was highly expressed in M1-NTRMs and was significantly negatively correlated with SNHG17 in TAMs.

To exclude the possibility that SNHG17 modulates the transcription of miR-628-5p and the synthesis of pri-miRNA or pre-miRNA, the activity of SNHG17 in the promoter region, pri-miRNA and pre-miRNA was evaluated. The results confirmed that SNHG17 did not affect these biological processes (Fig. 4f-h). Furthermore, both RIP and RNA pull-down assays demonstrated that SNHG17 and miR-628-5p could be significantly enriched in AGO2 within TAMs (Fig. 4i and S6c). Based on the predicted binding sites and the designed mutant plasmids, the interaction between SNHG17 and miR-628-5p was investigated through dual-luciferase reporter assays (Fig. 4j-k).

To delineate the relationship between miR-628-5p and the polarization and pro-tumorigenic function of M2-like macrophages, miR-628-5p inhibitor plasmids were transfected into SNHG17-knockdown THP-1 cells. qPCR and flow cytometry analysis determined that miR-628-5p inhibitor could up-regulate the expression of M2-like macrophage markers, especially in comparison with the SNHG17 sh1 group (Figure S6d-m). Additionally, after transfection with the miR-628-5p inhibitor, the suppressive effect of SNHG17 knockdown on tumor cell growth was substantially reversed, as evidenced by CCK-8, colony formation assays, and organoid proliferation assays (Figure S7a-j). Moreover, the migratory and invasive capacities of PANC-1 or PATU-8988 cells were restored following co-culture with THP-1 cells in the miR-628-5p inhibitor group (Figure S8a-c). Taken together, these findings demonstrated that miR-628-5p could inhibit the polarization of pro-tumor macrophages to suppress the malignant progression of PDAC.

Meanwhile, the ENCORI, miRWalk, and DIANA databases were queried to predict 12 potential downstream targets of miR-628-5p; the results yielded that only the expression of PGK1 was altered when miR-628-5p mimics or inhibitor plasmids were transfected in THP-1 cells (Fig. 4l, m and Figure S8d). Thereafter, the expression levels of PGK1 in 30 pairs of clinical samples were detected. The results showed that compared to M1-NTRMs, the expression level of PGK1 was enhanced in TAMs and was negatively correlated with miR-628-5p expression (Figure S8e, f). In addition, dual-luciferase reporter assays demonstrated that miR-628-5p could directly bind to PGK1 (Fig. 4n, o). Hence, the hypothesis that PGK1 is a downstream target of miR-628-5p was proposed. Intriguingly, PGK1 expression demonstrated a positive correlation with SNHG17 in TAMs from 30 paired clinical samples (Figure S8g). Previous studies suggested that PGK1 is an essential regulatory enzyme and impacts the polarization and function of TAMs by modulating aerobic glycolysis [35]. Western blotting assays demonstrated that knocking down SNHG17 down-regulated the expression of genes involved in aerobic glycolysis. However, upon transfection with miR-628-5p in TAMs, the expression levels of these genes were restored (Fig. 4p, q). In summary, SNHG17 acted as a ceRNA and modulated PGK1 expression by sponging miR-628-5p, enhancing the polarization and pro-tumor activities of M2-like macrophages.

### The binding of SNHG17 to the PGK1 protein in TAMs

**Fig. 5:**
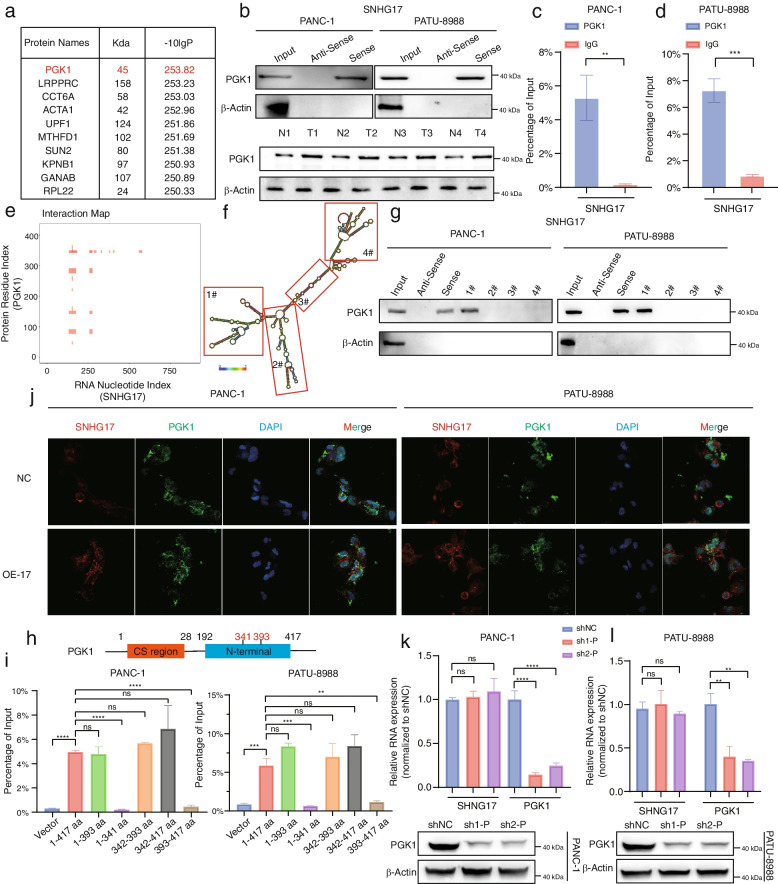
SNHG17 interacted with the PGK1 protein in THP-1 cell-derived TAMs. **A** RNA-binding proteins potentially binding to SNHG17 tapped by RNA pull-down experiments. **B** Up: Immunoblotting to validate the interaction between PGK1 protein and biotinylated SNHG17. Down: PGK1 protein expression level in TAMs and NTRMs from PDAC patients. (*n* = 4) (**C**-**D**) PGK1 antibody was used for RIP-qPCR to determine whether SNHG17 binds to PGK1 in THP-1 cell-derived TAMs (co-cultured with PANC-1 cells (**C**) and PATU-8988 cells (**D**)). **E** Prediction of SNHG17 and PGK1 protein binding regions by catRAPID. **F** SNHG17 secondary structure analyzed by RNAfold web server. The fragments of SNHG17 are shown in red boxes. **G** Immunoblot analysis of the ability of PGK1 to directly bind to biotinylated SNHG17 truncations. **H** Schematic representation of the structural domain of the PGK1 protein. **I** Flag RIP-qPCR analysis revealing binding levels of various truncations of PGK1 to SNHG17 in THP-1 cells co-cultured with PANC-1 cells or PATU-8988 cells. (**J**) Representative images of the colocalization of SNHG17 and PGK1 in THP-1 cell-derived TAMs. **K**-**L** WB and qPCR analyses of SNHG17 and PGK1 in THP-1 cells in which PGK1 was knocked down following co-culture with PANC-1 cells (**K**) or PATU-8988 cells (**L**). *P < 0.05; **P < 0.01; ***P < 0.001; ****P < 0.0001

Previous studies have insinuated that lncRNAs form complexes and function by interacting with cytoplasmic proteins [36]. RNA pull-down assay and mass spectrometry analysis revealed that PGK1 was one of the candidate RNA-binding proteins for SNHG17 in TAMs (Fig. 5a). Then, the formation of complexes via physical interactions between SNHG17 and the PGK1 protein (Fig. 5b) was validated. RIP-qPCR confirmed the interaction between SNHG17 and PGK1 (Fig. 5c, d). Notably, PGK1 was expressed at higher levels in TAMs than in M1-NTRMs (Fig. 5b). To further identify the specific binding regions of SNHG17 and the PGK1 protein, their potential binding regions were predicted using the catRAPID website (Fig. 5e). A series of truncated plasmids were designed based on the secondary structure predictions of SNHG17 from the RNAfold database (Fig. 5f). As illustrated in Fig. 5g, the 1–250 nt region of SNHG17 was the core region for direct binding. Furthermore, tagged truncated and full-length PGK1 mutants were transfected in HEK-293 T cells to determine the PGK1 domain primarily responsible for interacting with SNHG17. RIP assay findings demonstrated that the 341–393 segment of the N-terminal was essential for the binding of PGK1 to SNHG17 (Fig. 5h, i). Additionally, the IF assay revealed that SNHG17 was strongly co-expressed with PGK1 in the cytoplasm of THP-1 cell-derived TAMs, indicating a potential regulatory mechanism between SNHG17 and the PGK1 protein (Fig. 5j). At the same time, PCR and Western blotting assays determined that SNHG17 upregulated the expression of PGK1 at both the mRNA and protein levels (Figure S9a-f). Of note, the expression of SNHG17 remained largely unaltered regardless of PGK1 expression in THP-1 cells-derived TAMs (Fig. 5k, l and Figure S9g-i). Thus, SNHG17 was identified to be an upstream target of PGK1 and regulated PGK1 expression at both mRNA and protein levels in TAMs. To further elucidate the role of PGK1 in TAMs, qPCR and flow cytometry assays were performed, and the results were comparable to those with SNHG17 (Figure S10a-j). As displayed in Figure S10k-o and S11a-f, the loss of PGK1 could drastically suppress the proliferation of tumor cells. Moreover, the migratory and invasive abilities of PCs were evidently reduced when PGK1 was inhibited in TAM-derived THP-1 cells (Figure S12a-c). It was worthwhile emphasizing that the expression level of PGK1 in the SNHG17 sh1 group was lower than that in the control group in both the subcutaneous tumor and the liver model (Fig. 3l, m, r, s). Given the crucial role of PGK1 in aerobic glycolysis, fluctuations in glucose metabolism in TAMs were investigated. Following SNHG17 knockdown in TAMs, glucose uptake capacity and lactate secretion were evidently reduced, whilst transfection of miR-628-5p in THP-1 cell-derived TAMs restored the level of aerobic glycolysis (Fig. 2o-s, Figure S2e, f and Figure S8h-k). Consistently, the lack of PGK1 could inhibit aerobic glycolysis (Figure S12d-g). Altogether, these findings suggested that SNHG17 not only regulated the expression and function of PGK1 but also facilitated glucose metabolism and pro-tumorigenic macrophage polarization by physically binding to the PGK1 protein.

### SNHG17 Accelerated pro-tumor macrophage polarization by inducing T168A Phosphorylation of the PGK1 protein

**Fig. 6:**
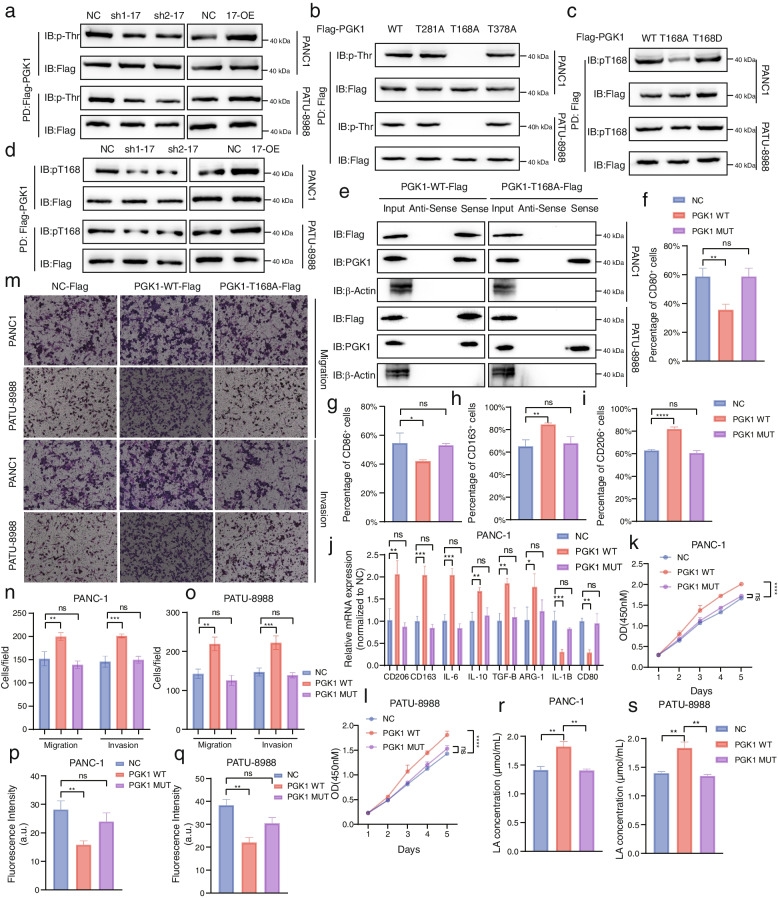
SNHG17 binding to PGK1 enhanced phosphorylation of the T168A site of PGK1 in THP-1 cell-derived TAMs. **A** and **D** THP-1 cells (shNC, sh1-SNHG17, sh2-SNHG17, NC, and SNHG17-OE) stably expressing Flag-PGK1 were co-cultured with PCs (PANC-1 or PATU-8988 cells) for 48 h. PD, pull-down. **B** Flag-PGK1 protein was pulled down from THP-1 cell-derived TAMs stably expressing Flag-PGK1 WT or PGK1 T281A, T168A, or T378A. **C** THP-1 cell-derived TAMs stably expressing Flag-PGK1 WT, PGK1 T168A, or PGK1 T168D were harvested. PGK1 pT168, phospho-PGK1 T168. **E** The binding ability of SNHG17 to PGK1 or mutated-PGK1 at the T168A site was analyzed by RNA pull-down assay. **F**-**I** Flow cytometric analysis of the expression of M1 markers (CD80 and CD86) and M2 markers (CD163 and CD206) in THP-1 cell-derived TAMs transfected with PGK1 overexpression plasmids or PGK1 mutated plasmids. **J** qPCR analysis of the expression of M2 markers and M1 markers in THP-1 cells co-cultured with PANC-1 cells (NC, PGK1-WT, PGK1-MUT). **K**-**L** CCK8 analysis of PANC-1 cells (**K**) or PATU-8988 cells (**L**) co-cultured with THP-1 cells transfected with PGK1 overexpression plasmids or mutated PGK1 plasmids. **M**–**O** Transwell assays were used to assess the migratory or invasive abilities of PANC-1 or PATU-8988 cells co-cultured with THP-1 cells. **P**-**S** Lactic acid concentration (**P**-**Q**) and glucose uptake (**R**-**S**) analysis of THP-1 cell-derived TAMs in each group (NC, PGK1-WT, and PGK1-MUT)

Protein phosphorylation, recognized as the most prevalent post-translational modification (PTM) in eukaryotes, can influence enzymatic activities to dictate their biological functions. This modulation is instrumental for protein function and participates in the progression of several tumors [37–39]. A previous study pointed out that phosphorylation modification is distinctly crucial for the PGK1 protein, accelerating the malignant development of glioblastoma by regulating the Warburg effect [35]. Therefore, the potential of SNHG17 to induce phosphorylation modification of PGK1 through physical binding was further evaluated. The phosphorylation level of FLAG-tagged PGK1 was examined in TAMs, and the results showed that SNHG17 knockdown could significantly suppress phosphorylation activity, whereas its overexpression manifested the opposite effect (Fig. 6a). Given that PGK1 can undergo phosphorylation at multiple sites, 3 potential phosphorylation sites of PGK1 in TAMs were predicted according to the reported literature and proteomic analyses of the PhosphoSitePlus and GeneCards databases [40, 41]. As shown in Figs. 6b and 6c, T168A was identified as the specific phosphorylation site on the PGK1 protein induced by SNHG17. To analyze the impact of SNHG17 on the phosphorylation level of T168A, immunoblotting(IB) was conducted, which confirmed that SNHG17 positively regulated the phosphorylation activity of T168A on PGK1 (Fig. 6d). Moreover, after transfecting T281A, T168A, and T378A mutants into TAMs, neither the T281A nor the T378A sites of PGK1 affected the binding of PGK1 to SNHG17, suggesting that the binding of PGK1 to SNHG17 also necessitates phosphorylation of the T168A site (Fig. 6e and Figure S13a, b). Subsequently, the relationship between M2-like macrophage polarization and the pro-tumorigenic function of PGK1 and the phosphorylation of the T168A site of PGK1 was further determined. The results concluded that mutation of the PGK1 T168A site significantly inhibited the polarization of M2-like macrophages (Fig. 6f-j and Figure S14a-e). Moreover, the phosphorylation of the T168A site was required for TAMs to exert pro-tumorigenic functions (Fig. 6k-o and Figure S14f-k). Notably, as corroborated by the glucose uptake and lactate production assays, glucose metabolism was similarly impaired by the mutation in the T168A site of PGK1 (Fig. 6p-s). Collectively, these data added to the pool of evidence that the phosphorylation of the PGK1 T168A site induced by SNHG17 was vital for PGK1 to enhance aerobic glycolysis and promote M2-like macrophage polarization.

### SNHG17 mediated the phosphorylation of PGK1 by recruiting the ERK1/2 Protein

**Fig. 7:**
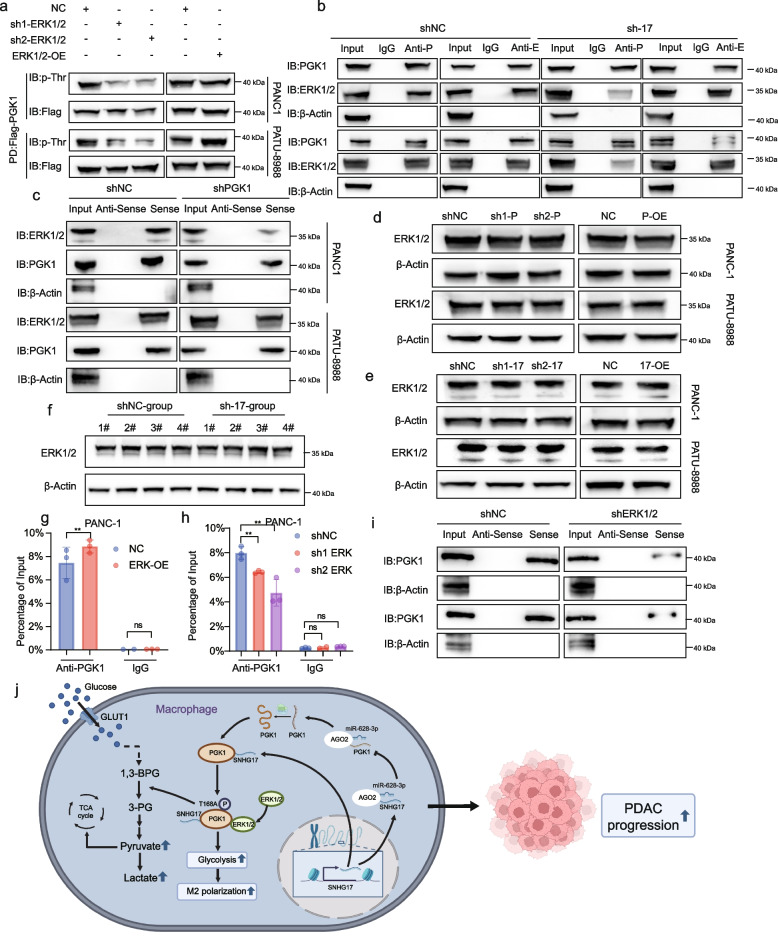
SNHG17 formation of SNHG17/PGK1/ERK1/2 complex promoted phosphorylation at the T168A site of PGK1. **A** The level of phosphorylation of PGK1 in THP-1 cell-derived TAMs (sh-ERK1/2 and ERK1/2-OE). **B**-**C** RNA pull-down and WB analysis of SNHG17 interacting with the ERK1/2 and PGK1 proteins in THP-1 cells transfected with shRNA of SNHG17 (**B**) and PGK1 (**C**). **D**-**E** The protein expression level of ERK1/2 in THP-1 cell-derived TAMs (sh-PGK1 and PGK1-OE (**D**) and sh-SNHG17 and SNHG17-OE (**E**)). **F** ERK1/2 expression levels in macrophages isolated from subcutaneous tumors (*n* = 4) (**G**-**H**) RIP-qPCR was utilized to identify the interaction between SNHG17 and PGK1 in THP-1 cells (ERK1/2 overexpression (**G**) and ERK1/2 knockdown (**H**)) co-cultured with PANC-1 or PATU-8988 cells. **I** Immunoblot analysis of the ability of PGK1 to bind directly to SNHG17 in THP-1 cell-derived TAMs after knockdown of ERK1/2. **J** Graphical summary of the major findings in this study

As an effector kinase in the MAPK signaling pathway, ERK1/2 occupies a central position in the cellular signal transduction network, which is capable of governing cellular life activities by phosphorylating substrates and regulating a variety of biological processes related to tumors, including cell proliferation, differentiation, migration, and angiogenesis [42–44]. Previous studies have pointed out that special-site phosphorylation of PGK1 depended on the recruitment and binding of ERK1/2 [45]. Thus, the hypothesis that SNHG17 could recruit ERK1/2 to promote PGK1 protein phosphorylation modification was considered. To confirm this hypothesis, the phosphorylation level of PGK1 in TAMs following ERK1/2 knockdown or overexpression was examined, and the results revealed that ERK1/2 considerably affected the phosphorylation modification of PGK1 (Fig. 7a). Subsequent analyses determined that upon SNHG17 knockdown, the binding capacity between PGK1 and ERK1/2 was diminished and vice versa, signifying that SNHG17 could recruit ERK1/2 to interact with PGK1 (Fig. 7b and Figure S15a). To identify the binding sites between ERK1/2 and PGK1, we utilized the series of PGK1 mutants and found that T168A was important for the PGK1-ERK1/2 interaction (Figure S15b). Meanwhile, RNA pull-down was performed after suppressing the expression of PGK1 in TAMs, and the results suggested that the ability of SNHG17 to bind to ERK1/2 was reduced, which supported the collaborative action between PGK1 and ERK1/2 (Fig. 7c). To exclude any possibility that SNHG17, PGK1, and ERK1/2 modulated the expression of each other, their individual mRNA and protein levels were examined. As expected, there was no upstream or downstream relationship between ERK1/2 and either PGK1 or SNHG17, which indicated that the ERK1/2 protein was solely recruited by SNHG17 (Fig. 7d-f and Figure S16a-g). To further elucidate the role of ERK1/2 in the interaction between SNHG17 and PGK1, knockdown or overexpressed plasmids of ERK1/2 were transfected into TAMs, and RNA pull-down assays were performed. As portrayed in Fig. 7g-i and Figure S16h-j, downregulation of ERK1/2 expression led to a decrease in the ability of SNHG17 to interact with the PGK1 protein, whereas overexpression of ERK1/2 had the opposite outcome, implying that ERK1/2 could enhance the binding activity between SNHG17 and the PGK1 protein, in line with the results of RIP assays.

In summary, these findings revealed that SNHG17 mediated the phosphorylation modification of PGK1 at the T168A site by recruiting ERK1/2, whilst ERK1/2 enhanced the physical binding of SNHG17 to PGK1.

## Discussion

PDAC is strikingly lethal due to its unique TME, wherein TAMs play a central role [46]. Over the past two decades, an increasing number of lncRNAs exerting a critical effect on the malignant progression of tumors have been identified [47–49]. The present study discovered that the expression level of SNHG17 was higher in TAMs derived from PDAC tissues compared to NTRMs from paracancerous tissues. Moreover, as a pro-oncogenic lncRNA in TAMs, SNHG17 activated M2 polarization and the malignant biological behavior of PCs, correlating with a poor prognosis in PDAC patients. Mechanistically, SNHG17 released PGK1 mRNA by sponging miR-628-5p. Subsequently, SNHG17, in conjunction with the translated PGK1 protein, enhanced the phosphorylation level at the T168A site of PGK1 by recruiting ERK1/2, ultimately altering glycolysis (Fig. 7j).

SNHG17, a member of the Small Nucleolar RNA Host Gene family, is aberrantly expressed in cancer cells from multiple tumor types, including colorectal, lung, and gastric cancer [26, 50, 51]. While the importance of lncRNAs in tumor-associated immune cells is frequently overlooked, existing studies indicated that the function of SNHG17 in the immune cells of TME remains ambiguous. Herein, lncRNA sequencing unveiled that SNHG17 expression was up-regulated in TAMs, as demonstrated by sorting of macrophages from PDAC tissues and healthy tissues, and this finding was subsequently corroborated by both qPCR and FISH analyses.

To fully comprehend the specific mechanisms of SNHG17 in TAMs, the miRNA (miR-628-5p) that SNHG17 potentially binds to was predicted using the online site TargetScan. Interestingly, miR-628-5p has been acknowledged as an oncogenic miRNA in various tumors, such as hepatocellular carcinoma, gastric carcinoma, and prostate carcinoma [52–54]. Wang et al. reported that miR-628-5p could be synthesized by M1 macrophages and was conducive to inhibiting liver cancer progression [53]. However, research addressing the role of miR-628-5p in PDAC remains scarce. Meanwhile, Lin et al. identified that miR-628-5p exerted oncogenic function by inhibiting the Akt and NF-kB pathways [55]. We postulate that miR-628-5p, an essential downstream member of SNHG17, regulates aerobic glycolysis in TAMs from PDAC by targeting the PGK1 mRNA to inhibit M2 polarization. The outcomes of this study evinced that the overall effects of miR-628-5p are comparable in the majority of tumors, but the underlying molecular mechanisms differ. Broader research efforts are necessitated to elucidate the mechanism of the function of miR-628-5p in tumors.

PGK1, a downstream target of miR-628-5p screened by various methods, has been widely reported to assume a decisive role in aerobic glycolysis in cancer cells [35, 56, 57]. Shao et al. found that PGK1 could alter the prognosis of PDAC by impacting the phosphorylation of PDHK1 [58]. At the same time, Zhang et al. explored the idea that IL-6 mediates the promotion of TAMs in tumor cells by modifying the phosphorylation of the PGK1 T243 site [26]. The present study uncovered that PGK1 protein could bind to SNHG17 and that PGK1 phosphorylation modification was regulated by the interaction between SNHG17 and PGK1. Phosphorylation of the T168A site of PGK1 was a cardinal factor affecting M2 polarization and the malignant biological behavior of PCs. Inhibitors targeting the phosphorylation of the PGK1 T168A site warrant further exploration.

The clinical application of lncRNA-targeted agents, including siRNA, ASO, and shRNA, remains limited for various diseases [59, 60]. Beyond issues related to the stability, specificity, and safety of biological agents, a possible cause might be that the identified functions of lncRNAs are not as impactful as anticipated. Overall, this study provided a novel direction and a theoretical basis for the identification of immunotherapeutic targets in pancreatic cancer treatment.

## Conclusions

This study uncovered the functions and mechanisms by which SNHG17 enhanced M2 polarization and promoted the growth and metastasis of PCs. SNHG17 sponged miR-628-5p to release PGK1 mRNA. Moreover, the binding of the PGK1 protein to SNHG17 recruited ERK1/2 and augmented the phosphorylation modification of T168A to reset aerobic glycolysis, signifying that SNHG17 is a candidate target for PDAC immunotherapy.

### Supplementary Information

**Additional file 1: Figure S1. **SNHG17 promotes M2 polarization and glucose uptake in THP-1 cells.

**Additional file 2: Figure S2. **SNHG17 in TAMs effects the proliferation of patient derived PDAC organoids.

**Additional file 3: Figure S3. **SNHG17 in BDT or MDT promoted M2 polarization.

**Additional file 4: Figure S4.** SNHG17 in BDT or MDT enhanced anaerobic glycolysis and promoted malignant progression of PCs.

**Additional file 5: Figure S5. **SNHG17 in BDT boosted growth and metastasis of PCs *in vivo*.

**Additional file 6: Figure S6. **SNHG17 sponges miR-628-5p to promote M2 polarization.

**Additional file 7: Figure S7. **SNHG17 sponges miR-628-5p to promote PDAC progression.

**Additional file 8: Figure S8. **SNHG17 sponges miR-628-5p to release PGK1 mRNA.

**Additional file 9: Figure S9. **SNHG17 interacts with PGK1 protein in THP-1 derived TAMs.

**Additional file 10: Figure S10. **PGK1 in THP-1 derived TAMs promotes M2 polarization and PDAC proliferation.

**Additional file 11: Figure S11. **PGK1 promotes the proliferation of patient derived PDAC organoids.

**Additional file 12: Figure S12. **PGK1 promotes migration, invasion, glucose uptake and LA release.

**Additional file 13: Figure S13.** Binding ability of SNHG17 to PGK1 or PGK1 mutations.

**Additional file 14: Figure S14. **SNHG17 in TAMs binds to PGK1 to promote M2 polarization and proliferation of PCs through T168A of PGK1.

**Additional file 15: Figure S15. **SNHG17 binds to PGK1.

**Additional file 16: Figure S16. **SNHG17 binds to PGK1 to enhance the phosphorylation of PGK1.

**Additional file 17: Table S1.** Clinicopathologic characteristics of 30 patients with PDAC from Ruijin Hospital.

**Additional file 18: Table S2.** Sequences of lentivirus targeting related genes.

**Additional file 19: Table S3.** Related primer sequences.

**Additional file 20: Table S4.** Antibodies for assays.

**Additional file 21: Table S5.** Clinicopathologic features of 96 patients with PDAC from Ruijin Hospital in tissue microarrays.

**Additional file 22.** Supplementary Table and Figure Legends

## Data Availability

Data are available in a public, open access repository. All sequencing data generated in this study are deposited at the National Omics Data Encyclopedia (NODE) with the accession code OEP019554.
