# Supplementary figures and images for: SNHG17 alters anaerobic glycolysis by resetting phosphorylation modification of PGK1 to foster pro-tumor macrophage formation in pancreatic ductal adenocarcinoma
Source: J Exp Clin Cancer Res. 2023 Dec 15;42:339. doi: 10.1186/s13046-023-02890-z (PMC10722693; doi:10.1186/s13046-023-02890-z)

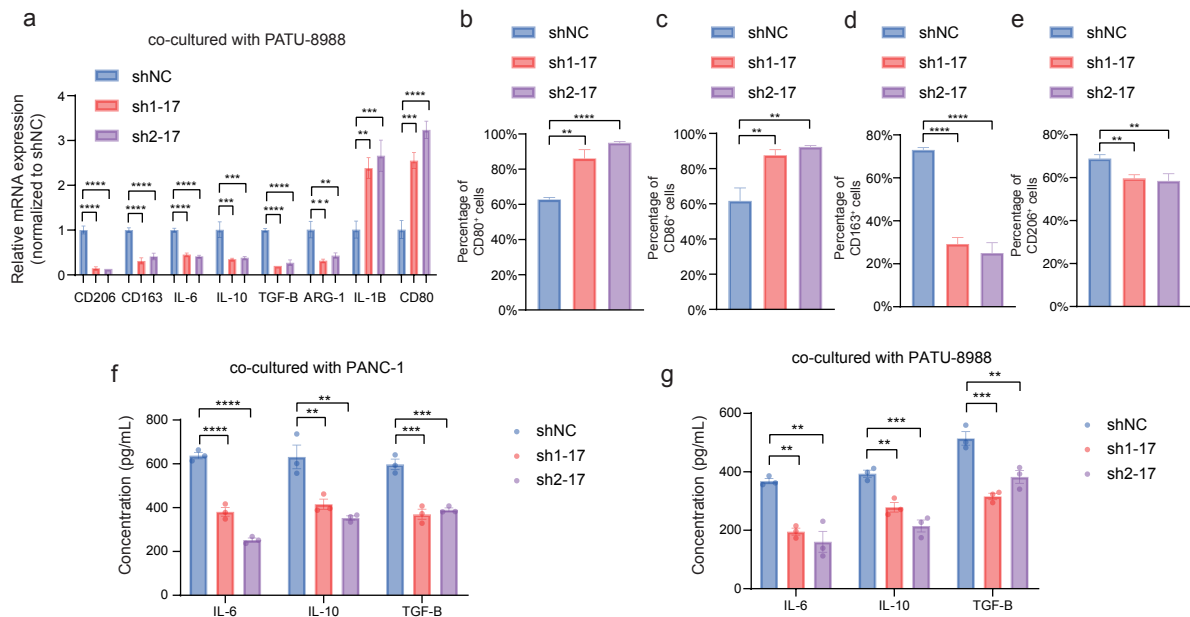

**Supplementary Figure 1**

Supplement: Supplementary file 1 — Additional file 1: Figure S1. SNHG17 promotes M2 polarization and glucose uptake in THP-1 cells. [file 13046_2023_2890_MOESM1_ESM.pdf]

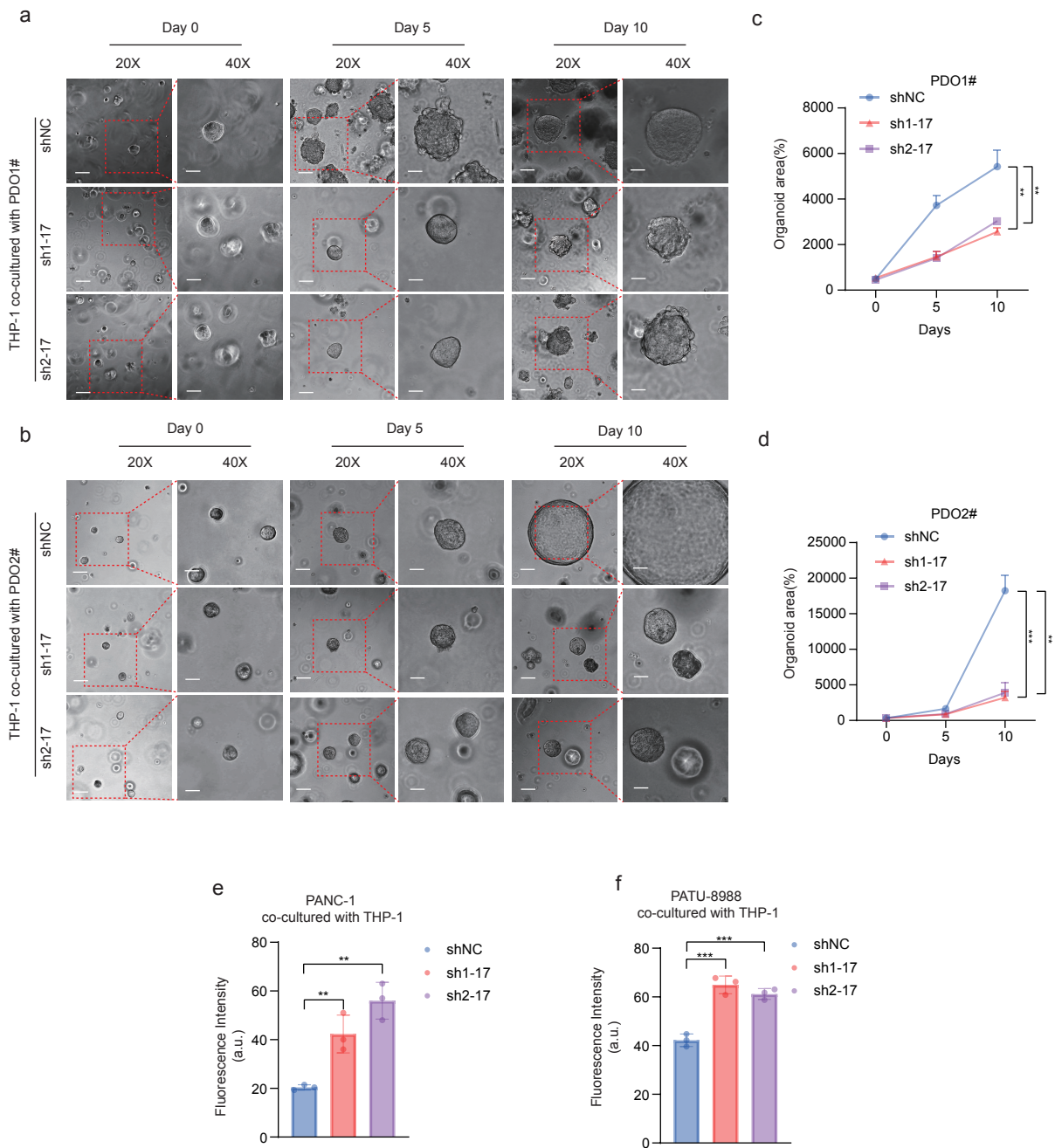

Supplementary Figure 2

Supplement: Supplementary file 2 — Additional file 2: Figure S2. SNHG17 in TAMs effects the proliferation of patient derived PDAC organoids. [file 13046_2023_2890_MOESM2_ESM.pdf]

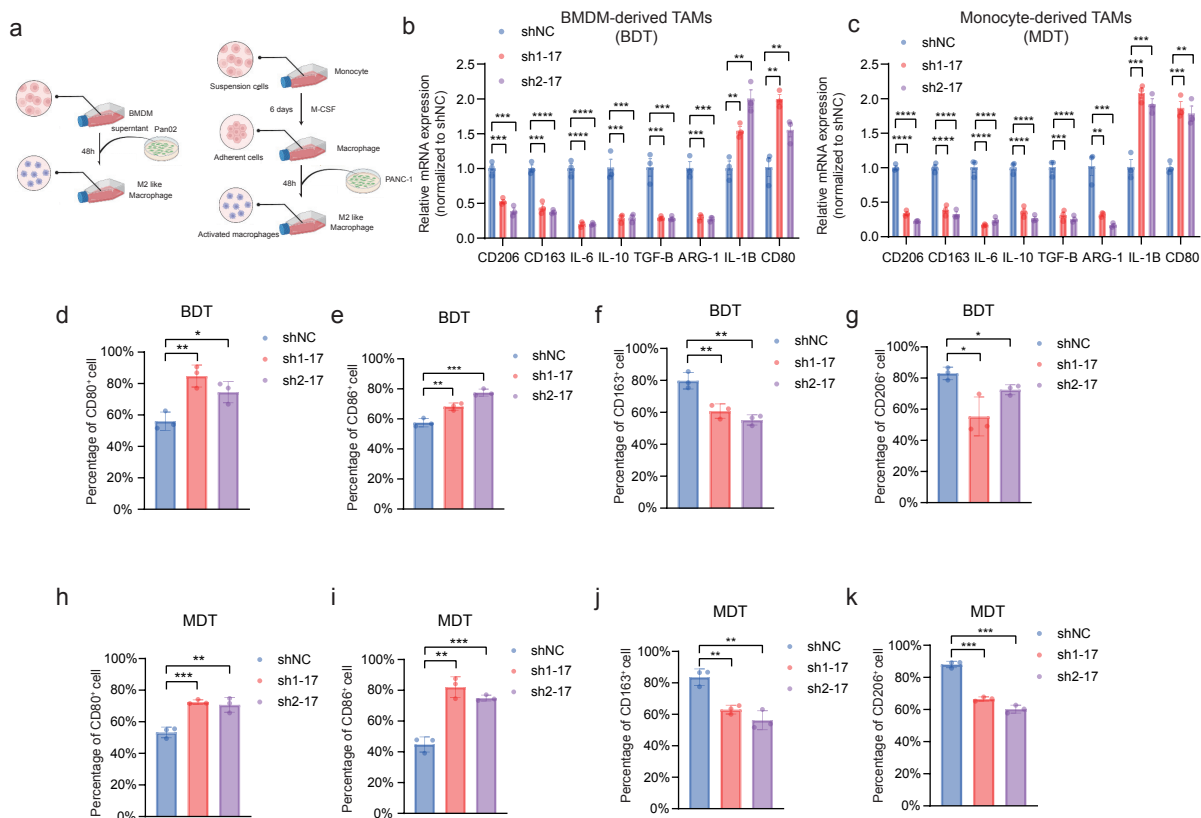

**Supplementary Figure 3**

Supplement: Supplementary file 3 — Additional file 3: Figure S3. SNHG17 in BDT or MDT promoted M2 polarization. [file 13046_2023_2890_MOESM3_ESM.pdf]

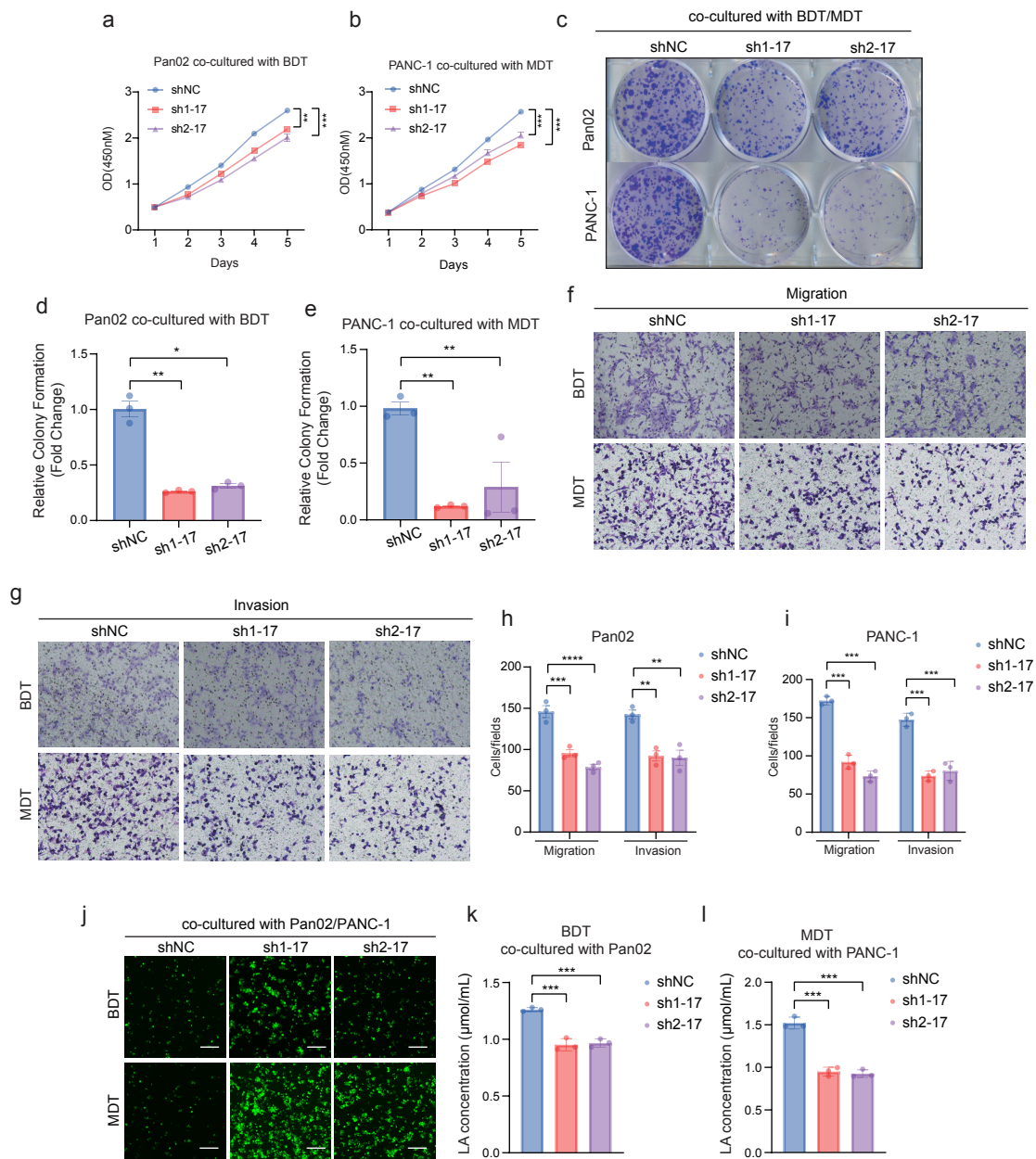

**Supplementary Figure 4**

Supplement: Supplementary file 4 — Additional file 4: Figure S4. SNHG17 in BDT or MDT enhanced anaerobic glycolysis and promoted malignant progression of PCs. [file 13046_2023_2890_MOESM4_ESM.pdf]

**a**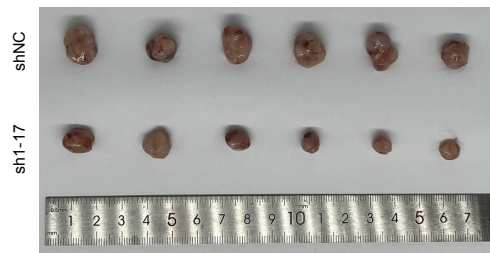**b**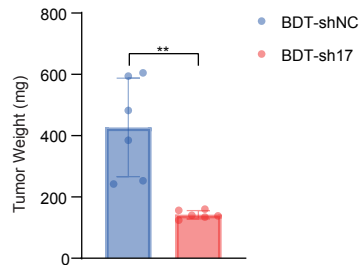**c**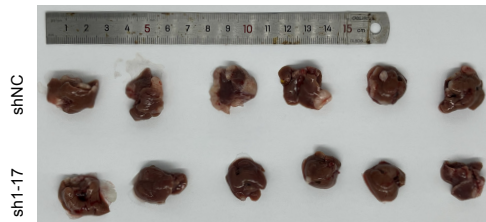**d**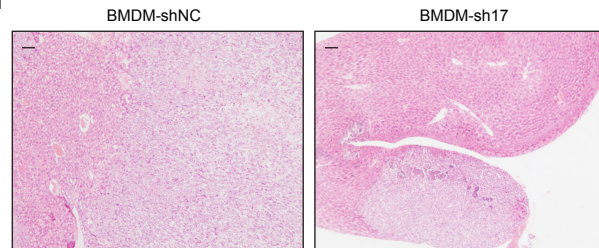

**Supplementary Figure 5**

Supplement: Supplementary file 5 — Additional file 5: Figure S5. SNHG17 in BDT boosted growth and metastasis of PCs in vivo. [file 13046_2023_2890_MOESM5_ESM.pdf]

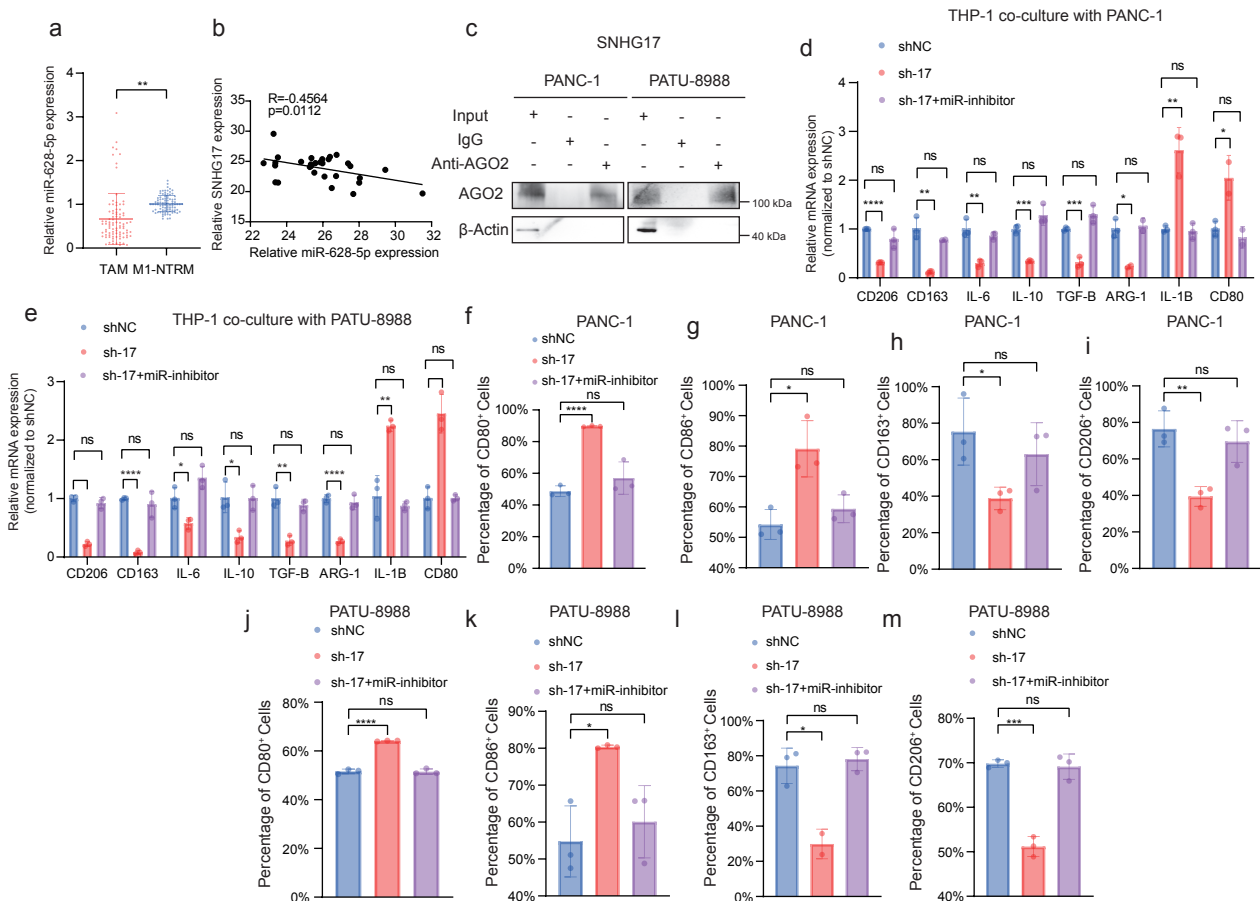

**Supplementary Figure 6**

Supplement: Supplementary file 6 — Additional file 6: Figure S6. SNHG17 sponges miR-628-5p to promote M2 polarization. [file 13046_2023_2890_MOESM6_ESM.pdf]

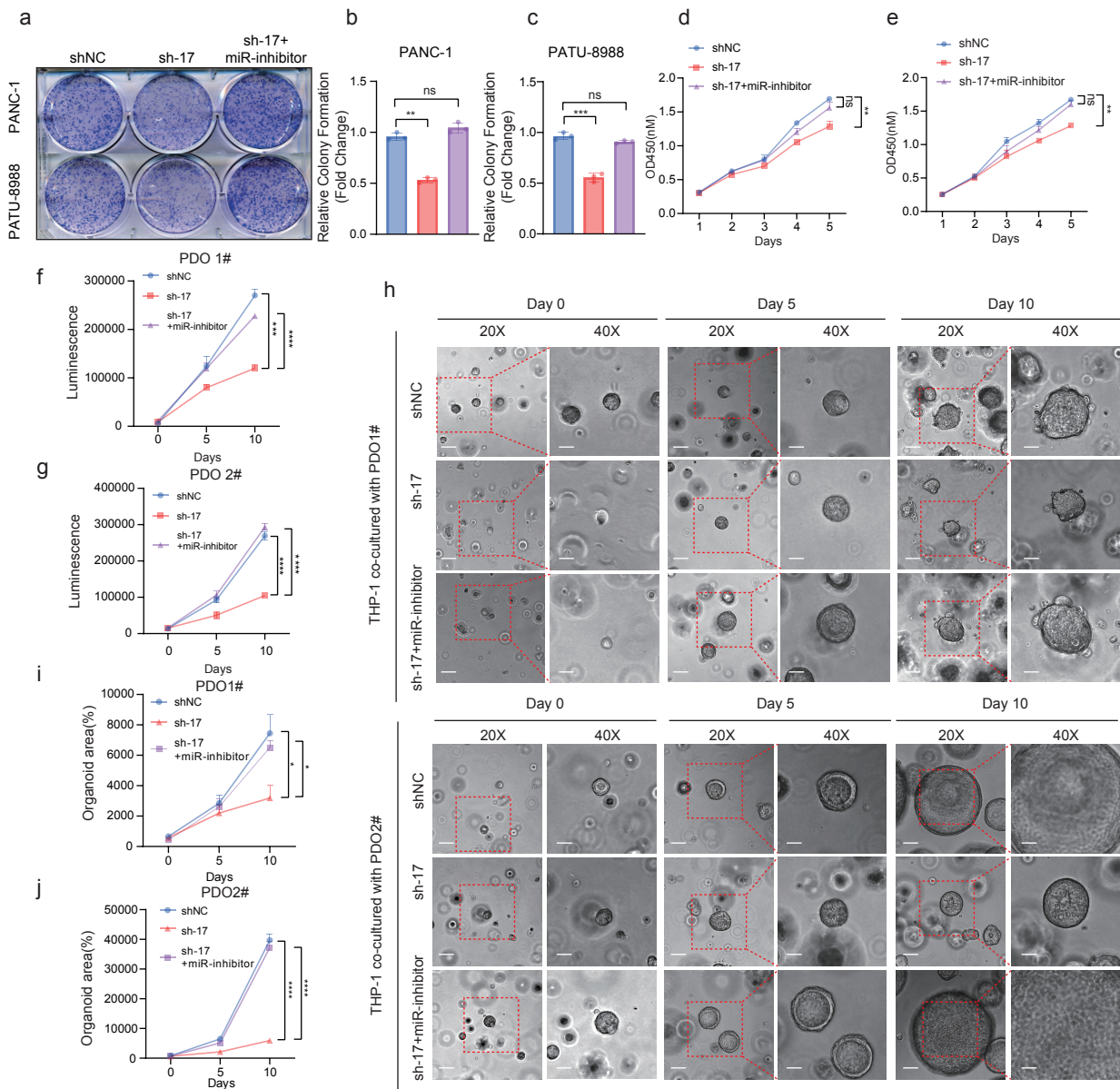

Supplementary Figure 7

Supplement: Supplementary file 7 — Additional file 7: Figure S7. SNHG17 sponges miR-628-5p to promote PDAC progression. [file 13046_2023_2890_MOESM7_ESM.pdf]

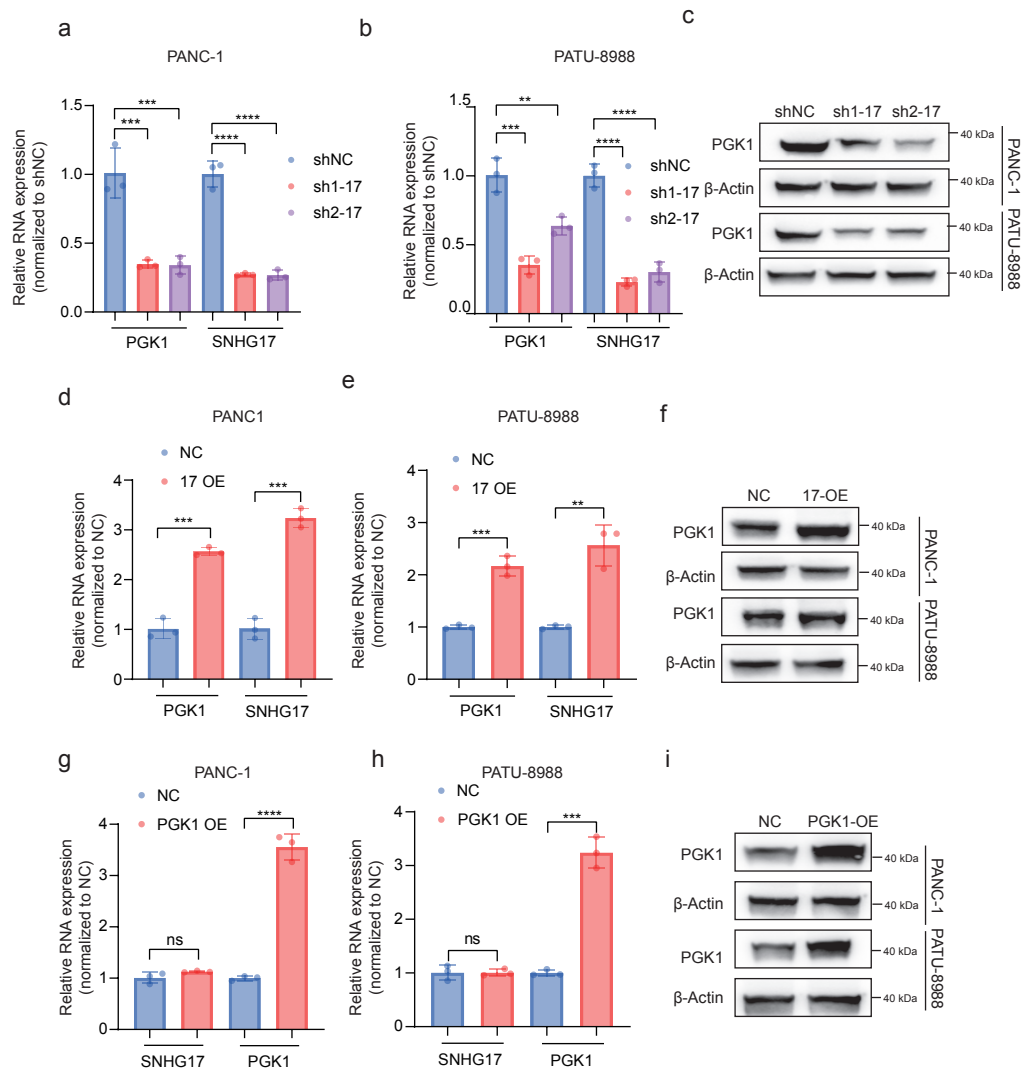

Supplement: Supplementary file 9 — Additional file 9: Figure S9. SNHG17 interacts with PGK1 protein in THP-1 derived TAMs. [file 13046_2023_2890_MOESM9_ESM.pdf]

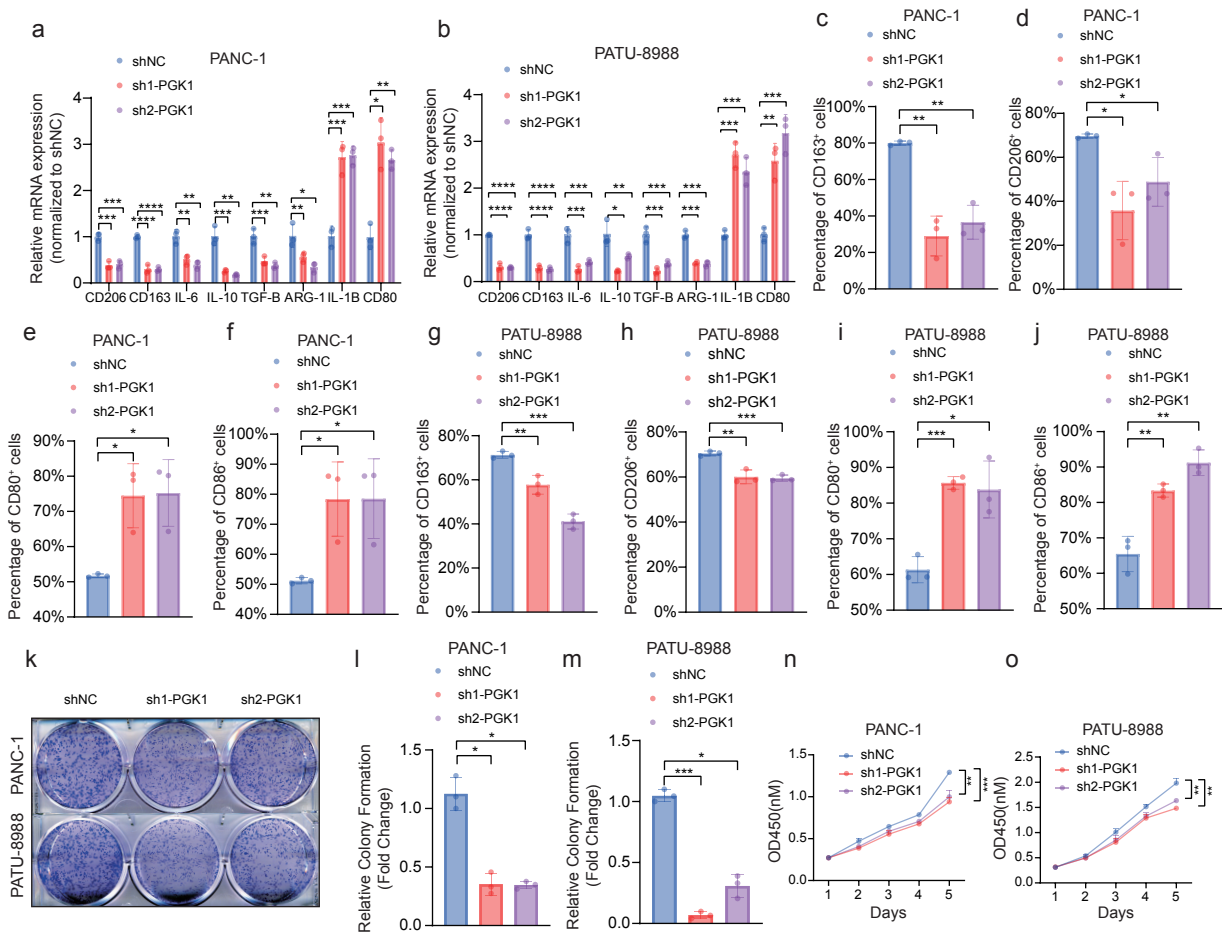

**Supplementary Figure 10**

Supplement: Supplementary file 10 — Additional file 10: Figure S10. PGK1 in THP-1 derived TAMs promotes M2 polarization and PDAC proliferation. [file 13046_2023_2890_MOESM10_ESM.pdf]

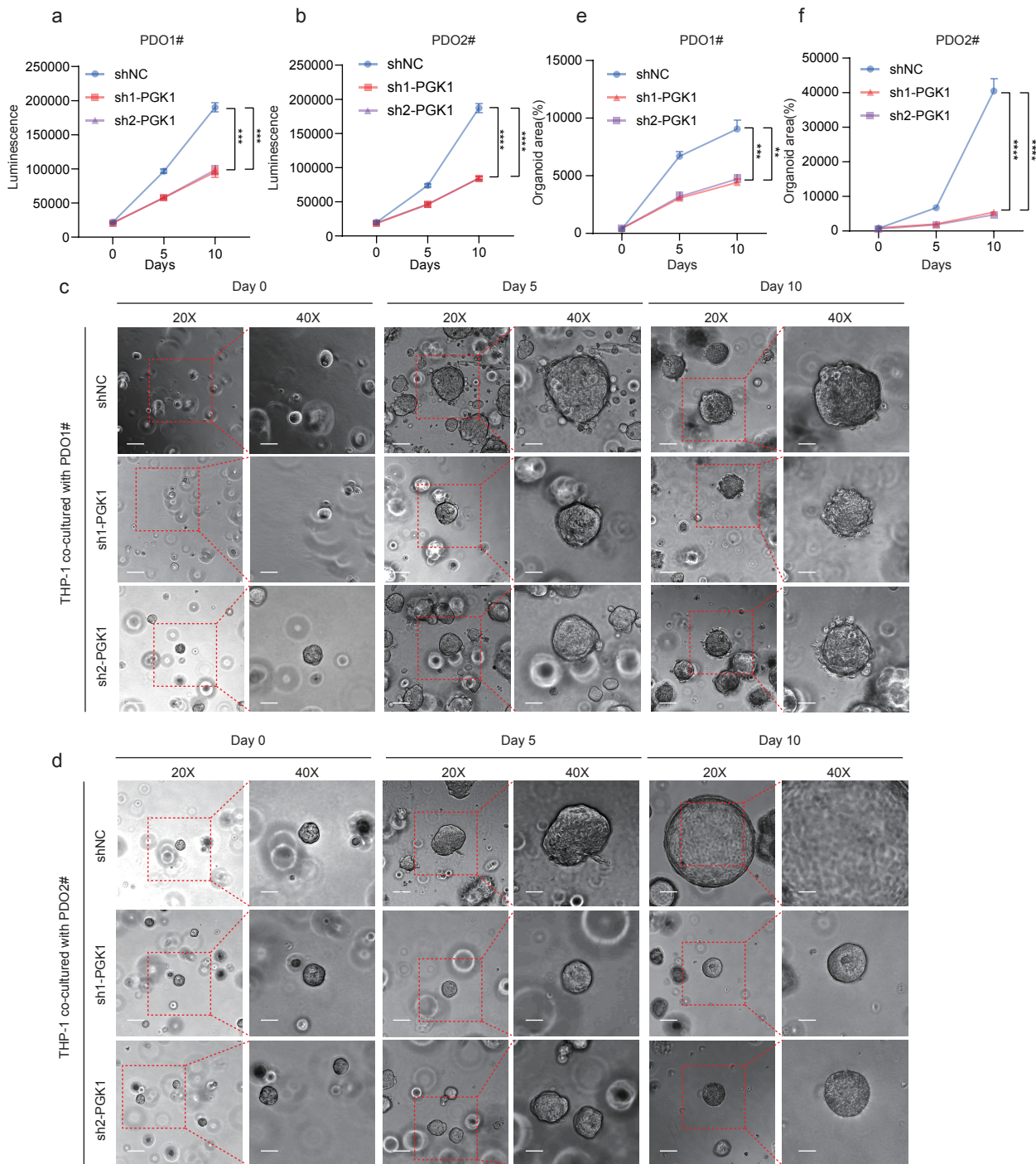

Supplementary Figure 11

Supplement: Supplementary file 11 — Additional file 11: Figure S11. PGK1 promotes the proliferation of patient derived PDAC organoids. [file 13046_2023_2890_MOESM11_ESM.pdf]

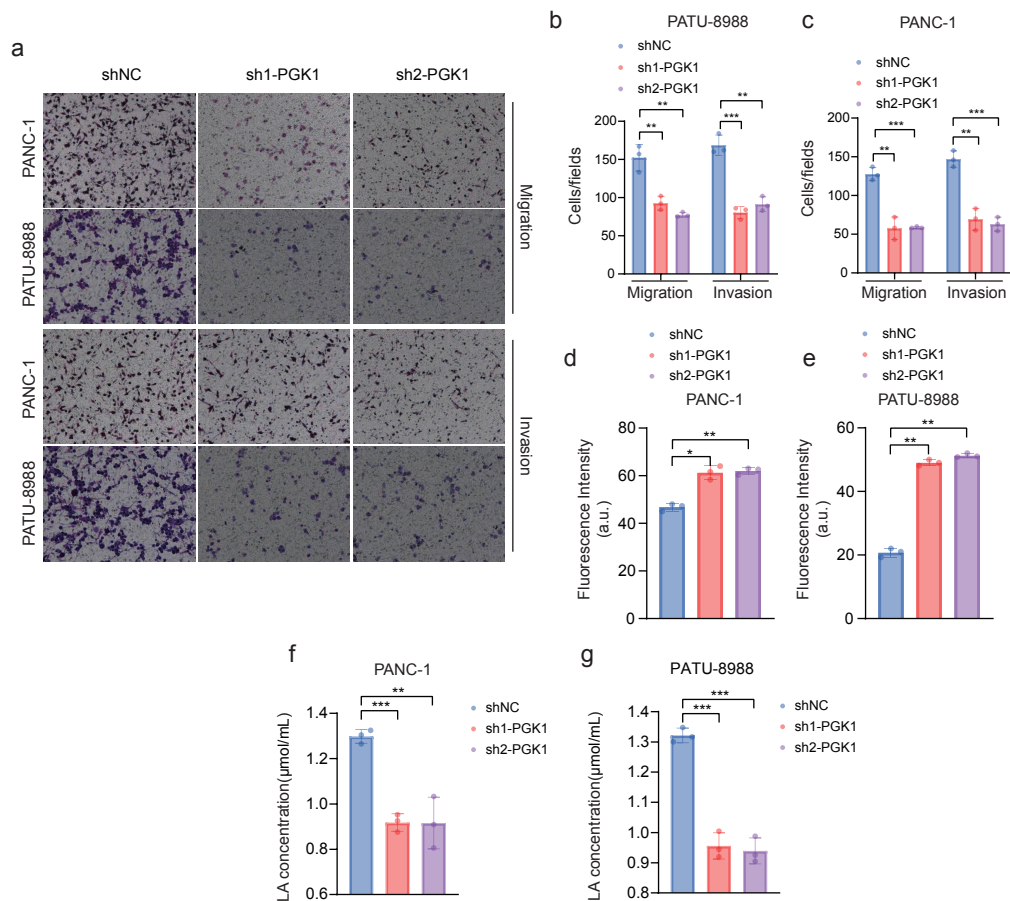

**Supplementary Figure 12**

Supplement: Supplementary file 12 — Additional file 12: Figure S12. PGK1 promotes migration, invasion, glucose uptake and LA release. [file 13046_2023_2890_MOESM12_ESM.pdf]

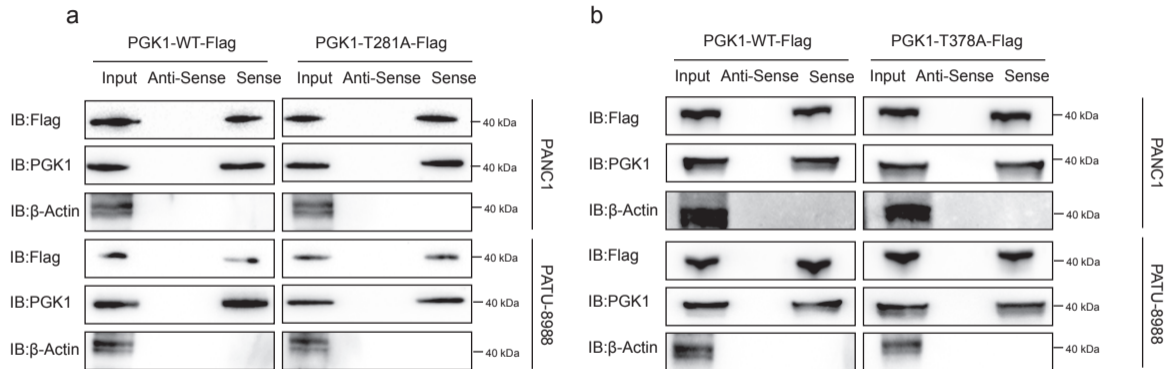

**Supplementary Figure 13**

Supplement: Supplementary file 13 — Additional file 13: Figure S13. Binding ability of SNHG17 to PGK1 or PGK1 mutations. [file 13046_2023_2890_MOESM13_ESM.pdf]

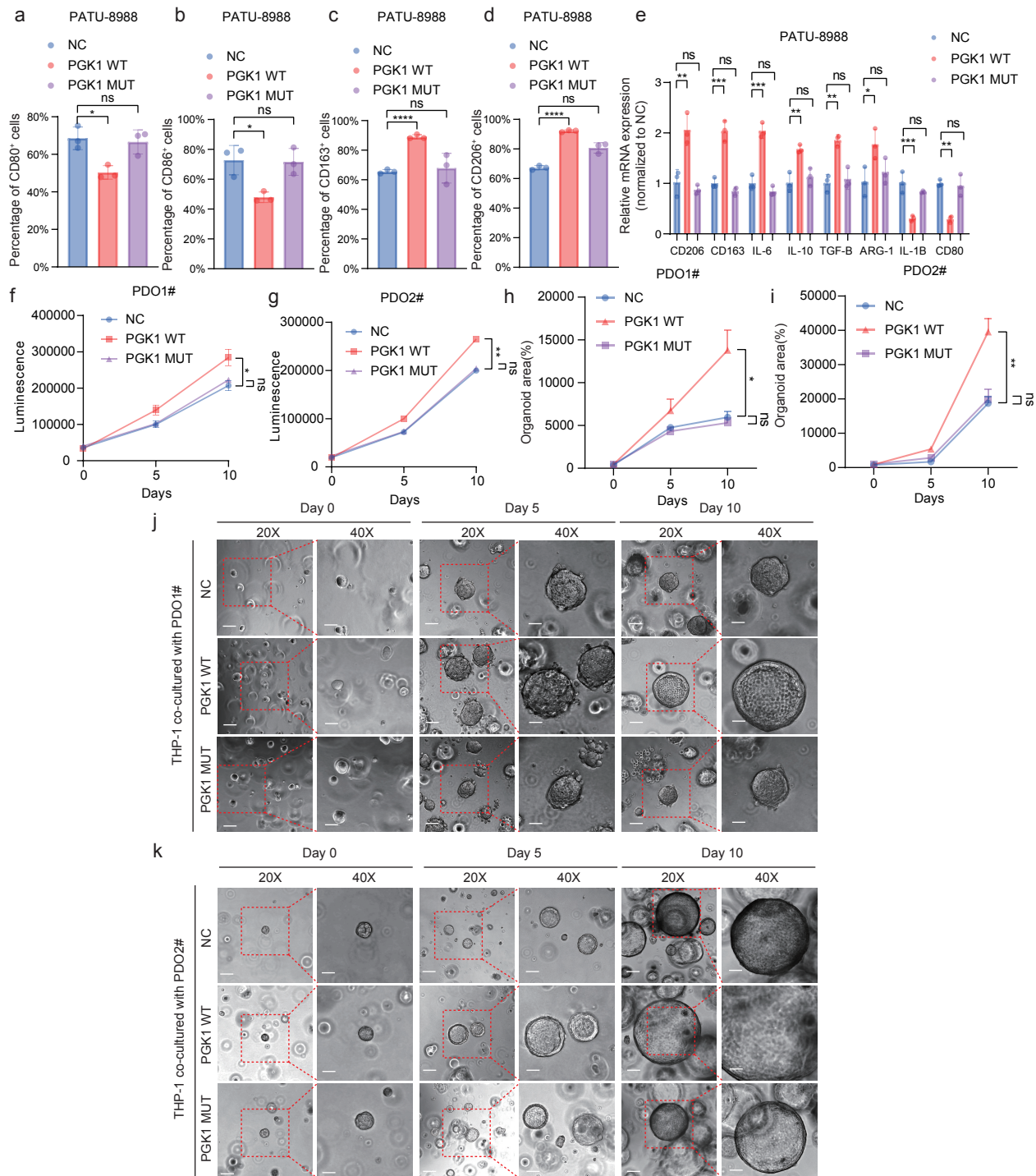

Supplementary Figure 14

Supplement: Supplementary file 14 — Additional file 14: Figure S14. SNHG17 in TAMs binds to PGK1 to promote M2 polarization and proliferation of PCs through T168A of PGK1. [file 13046_2023_2890_MOESM14_ESM.pdf]

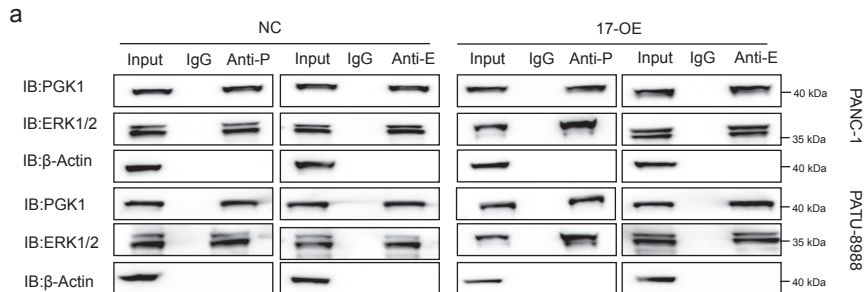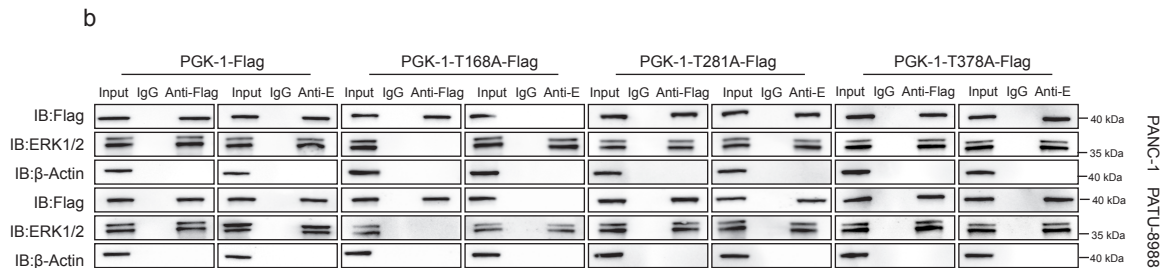

**Supplementary Figure 15**

Supplement: Supplementary file 15 — Additional file 15: Figure S15. SNHG17 binds to PGK1. [file 13046_2023_2890_MOESM15_ESM.pdf]

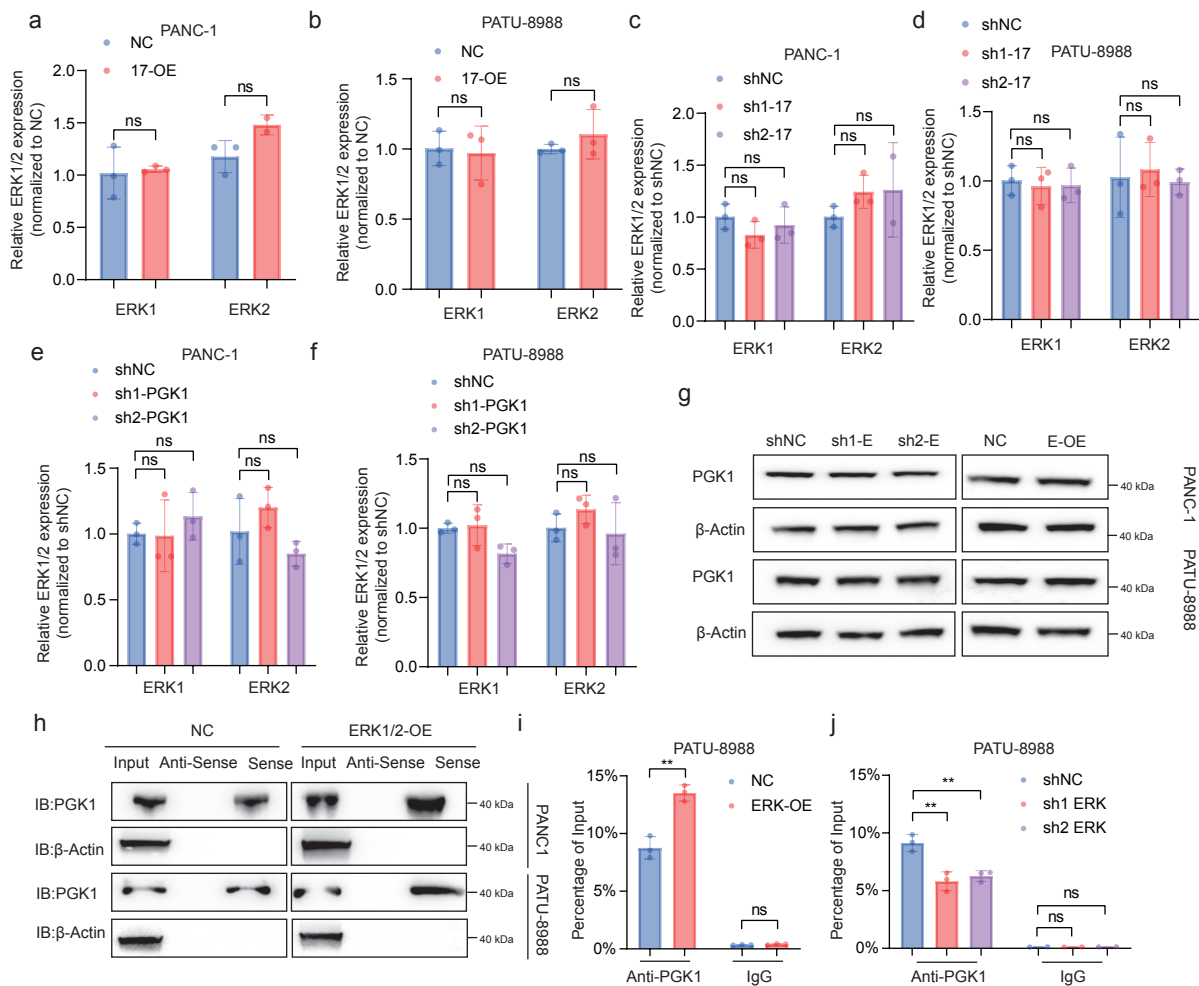

**Supplementary Figure 16**

Supplement: Supplementary file 16 — Additional file 16: Figure S16. SNHG17 binds to PGK1 to enhance the phosphorylation of PGK1. [file 13046_2023_2890_MOESM16_ESM.pdf]
