# Supplementary material for: SNHG17 alters anaerobic glycolysis by resetting phosphorylation modification of PGK1 to foster pro-tumor macrophage formation in pancreatic ductal adenocarcinoma
Source: J Exp Clin Cancer Res. 2023 Dec 15;42:339. doi: 10.1186/s13046-023-02890-z (PMC10722693; doi:10.1186/s13046-023-02890-z)
Supplement: Supplementary file 17 — Additional file 17: Table S1. Clinicopathologic characteristics of 30 patients with PDAC from Ruijin Hospital. [file 13046_2023_2890_MOESM17_ESM.docx]

**Table S1 Clinicopathologic characteristics of 30 patients with PDAC from Ruijin Hospital**

| **Characteristics** | **Number of cases** |
| --- | --- |
| **Gender** | |
| Female | 11 |
| Male | 19 |
| **Age (years)** | |
| < 60 | 12 |
| ≥ 60 | 18 |
| **Chemotherapy** |  |
| Yes | 3 |
| No | 27 |
| **AJCC stage** | |
| IA | 3 |
| IB  IIA | 6  4 |
| IIB | 15 |
| III  IV | 1  1 |
| **T classification** | |
| T1 | 5 |
| T2 | 14 |
| T3 | 11 |
| **N classification** | |
| N0 | 14 |
| N1 | 15 |
| N2 | 1 |
| **M classification** | |
| M0 | 0 |
| M1 | 30 |
| **LN metastasis** | |
| Positive | 13 |
| Negative | 17 |
|  |  |
|  | |
|  |  |
|  |  |
|  | |
|  |  |
