# Supplementary material for: SNHG17 alters anaerobic glycolysis by resetting phosphorylation modification of PGK1 to foster pro-tumor macrophage formation in pancreatic ductal adenocarcinoma
Source: J Exp Clin Cancer Res. 2023 Dec 15;42:339. doi: 10.1186/s13046-023-02890-z (PMC10722693; doi:10.1186/s13046-023-02890-z)
Supplement: Supplementary file 18 — Additional file 18: Table S2. Sequences of lentivirus targeting related genes. [file 13046_2023_2890_MOESM18_ESM.docx]

| **Table S2 Sequences of lentivirus targeting related genes** | | | | | |
| --- | --- | --- | --- | --- | --- |
| **ID** | **5’** | **stem** | **loop** | **stem** | **3’** |
| SNHG17-RNAi(sh1)-a | Ccgg | GGAGTTGGTGATCTGGGATCT | CTCGAG | AGATCCCAGATCACCAACTCC | TTTTTg |
| SNHG17-RNAi(sh1)-b | aattcaaaaa | GGAGTTGGTGATCTGGGATCT | CTCGAG | AGATCCCAGATCACCAACTCC |  |
| SNHG17-RNAi(sh2)-a | Ccgg | GCCTGGAATGACTTTAATAAC | CTCGAG | GTTATTAAAGTCATTCCAGGC | TTTTTg |
| SNHG17-RNAi(sh2)-b | aattcaaaaa | GCCTGGAATGACTTTAATAAC | CTCGAG | GTTATTAAAGTCATTCCAGGC |  |
| PGK1-RNAi(sh1)-a | Ccgg | GCCAAGATTGTCAAAGACCTA | CTCGAG | TAGGTCTTTGACAATCTTGGC | TTTTTg |
| PGK1-RNAi(sh1)-b | aattcaaaaa | GCCAAGATTGTCAAAGACCTA | CTCGAG | TAGGTCTTTGACAATCTTGGC |  |
| PGK1-RNAi(sh2)-a | Ccgg | GCCTACTTTATGGCAGACATT | CTCGAG | AATGTCTGCCATAAAGTAGGC | TTTTTg |
| PGK1-RNAi(sh2)-b | aattcaaaaa | GCCTACTTTATGGCAGACATT | CTCGAG | AATGTCTGCCATAAAGTAGGC |  |
| ERK1-RNAi(sh1)-a | Ccgg | GCAGCTGAGCAATGACCATAT | CTCGAG | ATATGGTCATTGCTCAGCTGC | TTTTTg |
| ERK1-RNAi(sh1)-b | aattcaaaaa | GCATATACAACCCGGAAAGAA | CTCGAG | ATATGGTCATTGCTCAGCTGC |  |
| ERK1-RNAi(sh2)-a | Ccgg | CGACCTTAAGATTTGTGATTT | CTCGAG | AAATCACAAATCTTAAGGTCG | TTTTTg |
| ERK1-RNAi(sh2)-b | aattcaaaaa | CGACCTTAAGATTTGTGATTT | CTCGAG | AAATCACAAATCTTAAGGTCG |  |
| ERK2-RNAi(sh1)-a | Ccgg | CAAAGTTCGAGTAGCTATCAA | CTCGAG | TTGATAGCTACTCGAACTTTG | TTTTTg |
| ERK2-RNAi(sh1)-b | aattcaaaaa | CAAAGTTCGAGTAGCTATCAA | CTCGAG | TTGATAGCTACTCGAACTTTG |  |
| ERK2-RNAi(sh2)-a | Ccgg | TATTACGACCCGAGTGACGAG | CTCGAG | CTCGTCACTCGGGTCGTAATA | TTTTTg |
| ERK2-RNAi(sh2)-b | aattcaaaaa | TATTACGACCCGAGTGACGAG | CTCGAG | CTCGTCACTCGGGTCGTAATA |  |
