# Supplementary material for: SNHG17 alters anaerobic glycolysis by resetting phosphorylation modification of PGK1 to foster pro-tumor macrophage formation in pancreatic ductal adenocarcinoma
Source: J Exp Clin Cancer Res. 2023 Dec 15;42:339. doi: 10.1186/s13046-023-02890-z (PMC10722693; doi:10.1186/s13046-023-02890-z)
Supplement: Supplementary file 19 — Additional file 19: Table S3. Related primer sequences. [file 13046_2023_2890_MOESM19_ESM.docx]

**Table S3 Related primer sequences**

| **Primers for RNA quantitation** | | |
| --- | --- | --- |
| Gene Symbol | Forward Sequence | Reverse Sequence |
| β-actin mRNA | GTCATTCCAAATATGAGATGCGT | GCTATCACCTCCCCTGTGTG |
| U6  mRNA | CTCGCTTCGGCAGCACA | AACGCTTCACGAATTTGCGT |
| CD206 mRNA | TCCGGGTGCTGTTCTCCTA | CCAGTCTGTTTTTGATGGCACT |
| CD163 mRNA | GACGCATTTGGATGGATCATGT | CCCACCGTCCTTGGAATTTGA |
| IL-6  mRNA | ACTCACCTCTTCAGAACGAATTG | CCATCTTTGGAAGGTTCAGGTTG |
| IL-10 mRNA | TCAAGGCGCATGTGAACTCC | GATGTCAAACTCACTCATGGCT |
| TGF-β  mRNA | CTAATGGTGGAAACCCACAACG | TATCGCCAGGAATTGTTGCTG |
| Arginase-1 mRNA | TGGACAGACTAGGAATTGGCA | CCAGTCCGTCAACATCAAAACT |
| IL-1β mRNA | AGCTACGAATCTCCGACCAC | CGTTATCCCATGTGTCGAAGAA |
| CD80 mRNA | AAACTCGCATCTACTGGCAAA | GGTTCTTGTACTCGGGCCATA |
| SNHG17 mRNA | AGGGGAAGCAAGGTGAAAGT | ATCCCAGATCACCAACTCCA |
| PGK1 mRNA | TGGACGTTAAAGGGAAGCGG | GCTCATAAGGACTACCGACTTGG |
| POLK mRNA | ACTTTGACAAATACCGAGCTGTG | GGAGAGATGGATCGTTCATGC |
| TMEM33  mRNA | CCTCTAGAAGAATTCCATATTGTCG | TGGAGACATAGTCTTCTCACAAACA |
| FLNB  mRNA | GCCTGTGGATAATGCACGAGA | GGCTCGATTCCTCTGCCATA |
| FAM168B mRNA | TCTGGGGTTCCCTATGCAAAT | GTAGGATTCGCTCCAGGATACA |
| PPHLN1 mRNA | GAGACGATCATTCTGCAAGCA | TCTCTCGCATAATGGGAAGAGTA |
| EIF2B1 mRNA | AGGGTCTGAGGGCGAATCTC | CAGGGAGGCAAGACTGATGAA |
| WNK1 mRNA | GTTAGCGCACACTCACTAGAT | CCCACCAGGCTGAGAAATATAA |
| EFR3A mRNA | ATGCCTACCCGAGTATGCTG | GGTAAGAACCAATTCGATCCAGT |
| ZC3H6 mRNA | AGGGCACGACAGAGAAGATG | AAGGCTGTAGTCCGAACTATCA |
| PIGS  mRNA | GCGGCTACACACCTAGAGG | CTGGGAGTAAGGCAACGAGG |
| SYNCRIP mRNA | CTGGTCTCAATAGAGGTTATGCG | TCCGGTTGGTGGTATAAAATGAC |
| MAPK1- mRNA | CGTGTTGCAGATCCAGACCA | GCCTGTTCCATGGCACCTTA |
| MAPK3-mRNA | ATGTCATCGGCATCCGAGAC | GGATCTGGTAGAGGAAGTAGCA |
