# Supplementary material for: SNHG17 alters anaerobic glycolysis by resetting phosphorylation modification of PGK1 to foster pro-tumor macrophage formation in pancreatic ductal adenocarcinoma
Source: J Exp Clin Cancer Res. 2023 Dec 15;42:339. doi: 10.1186/s13046-023-02890-z (PMC10722693; doi:10.1186/s13046-023-02890-z)
Supplement: Supplementary file 20 — Additional file 20: Table S4. Antibodies for assays. [file 13046_2023_2890_MOESM20_ESM.docx]

| **Table S4 Antibodies for assays** | | | |
| --- | --- | --- | --- |
| **Antibody** | **Company** | **Cat. No.** | **Species** |
| PGK1 | Abclonal | A12686 | Rabbit |
| [β-Actin](https://baike.sogou.com/v53215227.htm) | Abclonal | AC038 | Rabbit |
| GLUT1 | Abclonal | A11208 | Rabbit |
| ENO1 | Abclonal | A16841 | Rabbit |
| HK2 | Abclonal | A0994 | Rabbit |
| LDHA | Abclonal | A1146 | Rabbit |
| FLAG | Cell Signaling | 14793S | Rabbit |
| ERK1/2 | Abclonal | A16686 | Rabbit |
| p-Thr | Santa Cruz Biotechnology | sc-5267 | Mouse |
| CD206-PE | BioLegend | 321106 | Mouse-anti-human |
| CD163-PE-Cy7 | BioLegend | 333614 | Mouse-anti-human |
| CD80-PE-Cy7 | BioLegend | 305218 | Mouse-anti-human |
| CD86-APC | BioLegend | 305412 | Mouse-anti-human |
