# Supplementary material for: SNHG17 alters anaerobic glycolysis by resetting phosphorylation modification of PGK1 to foster pro-tumor macrophage formation in pancreatic ductal adenocarcinoma
Source: J Exp Clin Cancer Res. 2023 Dec 15;42:339. doi: 10.1186/s13046-023-02890-z (PMC10722693; doi:10.1186/s13046-023-02890-z)
Supplement: Supplementary file 21 — Additional file 21: Table S5. Clinicopathologic features of 96 patients with PDAC from Ruijin Hospital in tissue microarrays. [file 13046_2023_2890_MOESM21_ESM.docx]

**Table S5 Clinicopathologic features of 96 patients with PDAC from Ruijin Hospital in tissue microarrays.**

| **Characteristics** | **Number of cases** |
| --- | --- |
| **Gender** | |
| Female | 58 |
| Male | 38 |
| **Age (years)** | |
| < 60 | 34 |
| ≥ 60 | 62 |
| **AJCC stage** | |
| IB  IIA | 1  36 |
| IIB | 37 |
| III  IV | 12  10 |
| **T classification** | |
| T2 | 1 |
| T3 | 79 |
| T4 | 16 |
| **N classification** | |
| N0 | 45 |
| N1 | 51 |
| **M classification** | |
| M0 | 86 |
| M1 | 10 |
| **Survival Status** | |
| Alive | 31 |
| Dead | 65 |
|  |  |
