# Supplementary material for: SNHG17 alters anaerobic glycolysis by resetting phosphorylation modification of PGK1 to foster pro-tumor macrophage formation in pancreatic ductal adenocarcinoma
Source: J Exp Clin Cancer Res. 2023 Dec 15;42:339. doi: 10.1186/s13046-023-02890-z (PMC10722693; doi:10.1186/s13046-023-02890-z)
Supplement: Supplementary file 22 — Additional file 22. Supplementary Table and Figure Legends [file 13046_2023_2890_MOESM22_ESM.docx]

**Supplementary Table Legends**

**Table S1** Clinicopathologic characteristics of 30 patients with PDAC from Ruijin Hospital.

**Table S2** Sequences of lentivirus targeting related genes.

**Table S3** Related primer sequences.

**Table S4** Antibodies for assays.

**Table S5** Clinicopathologic features of 96 patients with PDAC from Ruijin Hospital in tissue microarrays.

**Supplementary Figure Legends**

**Figure S1 SNHG17 promotes M2 polarization and glucose uptake in THP-1 cells.**

(A) The mRNA expression of M2 markers (CD206, CD163, IL6, IL10, TGFB and Arg1) and M1 markers (IL1B and CD80) after knockdown of SNHG17 in THP-1 and co-culture with PATU-8988. (B-E) Flow Cytometry analysis of CD80 (B), CD86 (C), CD163 (D), CD206 (E) expression in THP-1 derived TAMs co-cultured with PATU-8988. (F-G) ELISA analysis of IL-6, IL-10 and TGFB in THP-1 derived TAMs. *P < 0.05; **P < 0.01; ***P < 0.001; ****P < 0.0001.

**Figure S2 SNHG17 in TAMs effects the proliferation of patient derived PDAC organoids.**

(A-B) Representative pictures of PDO1#(A) and PDO2#(B) after co-cultured with TAMs (shNC, sh1-SNHG17, sh2-SNHG17) in day0, day5 and day10. (C-D) Relative areas of co-cultured PDO1# (C) and PDO2# (D) in day0, day5 and day10. (E-F) Relative fluorescence intensity reflecting the capacity of glucose uptake in THP-1 co-cultured with PANC-1 (E) and PATU-8988 (F). *P < 0.05; **P < 0.01; ***P < 0.001; ****P < 0.0001.

**Figure S3 SNHG17 in BDT or MDT promoted M2 polarization.**

(A) Schematic representation of induction of BMDM (left) and monocytes (right) into TAMs *in vitro*. (B-C) The mRNA expression of M2 markers (CD206, CD163, IL6, IL10, TGFB and Arg1) and M1 markers (IL1B and CD80) after knockdown of SNHG17 in BDT (B) and MDT (C). (D-G) Flow Cytometry ananlysis of CD80/CD86/CD163/CD206 expression in BDT (shNC, sh1-SNHG17 and sh2-SNHG17). (H-K) Flow Cytometry ananlysis of CD80/CD86/CD163/CD206 expression in MDT (shNC, sh1-SNHG17 and sh2-SNHG17).

**Figure S4 SNHG17 in BDT or MDT enhanced anaerobic glycolysis and promoted malignant progression of PCs.**

(A-B) CCK8 analysis of Pan02 co-cultured with BDT (A) and PANC-1 co-cultured with MDT (B). (C-E) Representative colony formation images (C) of Pan02 co-cultured with BDT (shNC, sh1-SNHG17 and sh2-SNHG17) and PANC-1 co-cultured with MDT (shNC, sh1-SNHG17 and sh2-SNHG17). And colony numbers of Pan02 and PANC-1 in each group. (F-G) Representative migration (F) and invasion (G) images of Pan02 co-cultured with BDT (shNC, sh1-SNHG17 and sh2-SNHG17) and PANC-1 co-cultured with MDT (shNC, sh1-SNHG17 and sh2-SNHG17). (H-I) Migrated and invaded Pan02 cells (H) per field and PANC-1 cells (I) per field in each group (shNC, sh1-SNHG17 and sh2-SNHG17). (J-L) Glucose uptaking (J) and Lactic acid concentration (K, L) analysis of BDT and MDT in each group (shNC, sh1-SNHG17 and sh2-SNHG17).

**Figure S5** **SNHG17 in BDT boosted growth and metastasis of PCs *in vivo*.**

(A) Images of tumors from C57BL/6 mice which were injected with BDT (shNC and sh1-SNHG17) and Pan02 cells. (B) Weights of subcutaneous tumors from C57BL/6 mice in each group. (n=6) (C) Images of livers from C57BL/6 mice co-injected with BDT cells and Pan02 cells in the spleen. (D) HE images of the livers. Scar bar: 50μm.

**Figure S6 SNHG17 sponges miR-628-5p to promote M2 polarization.**

(A) Relative expression of miR-628-5p in TAM and M1-NTRM. (B) Relationship between the expressions of SNHG17 and miR-628-5p. (C) RIP analysis of THP-1 derived TAMs. (D-E) The mRNA expression of M2 markers (CD206, CD163, IL6, IL10, TGFB and Arg1) and M1 markers (IL1B and CD80) in TAMs after co-culture with PANC-1 (D) and PATU-8988 (E). (F-M) Flow Cytometry analysis of CD80, CD86, CD163, CD206 expression in THP-1 derived TAMs co-cultured with PANC-1 (F-I) and PATU-8988 (J-M). *P < 0.05; **P < 0.01; ***P < 0.001; ****P < 0.0001.

**Figure S7 SNHG17 sponges miR-628-5p to promote PDAC progression.**

(A) Colony assay of PDAC cell lines co-cultured with TAMs (shNC, sh-SNHG17 and sh-SNHG17+miR-628-5p inhibitor), and corresponding statistical figure of PANC-1 (B) and PATU-8988 (C). CCK8 assays of PANC-1 (D) and PATU-8988 (E) cells co-cultured with TAMs (shNC, sh-SNHG17 and sh-SNHG17+miR-628-5p inhibitor). (F-G) CTG analysis of two patient-derived organoids (PDO1# and PDO2#) co-cultured with TAMs. (H) Representative pictures of PDO1# and PDO2# after co-cultured with THP-1 derived TAMs (shNC, sh-SNHG17 and sh-SNHG17+miR-628-5p inhibitor) in day0, day5 and day10. (I-J) Relative areas of co-cultured PDO1# (I) and PDO2# (J) in day0, day5 and day10. *P < 0.05; **P < 0.01; ***P < 0.001; ****P < 0.0001.

**Figure S8 SNHG17 sponges miR-628-5p to release PGK1 mRNA.**

(A) Representative migration and invasion images of pancreatic cancer cells co-cultured with THP-1 derived TAMs (shNC, sh-SNHG17 and sh-SNHG17+miR-628-5p inhibitor). (B, H, J) Migrated and invaded cells of PANC-1, LA concentration and glucose uptake after co-cultured with TAMs. (C, I, K) Migrated and invaded cells of PATU-8988, LA concentration and glucose uptake after co-cultured with TAMs. (D) Relative mRNA expression of THP-1 derived TAMs. (E) Relative mRNA expression of PGK1 in TAM and M1-NTRM. (F) Relationship between the expressions of PGK1 and miR-628-5p. (G) Relationship between the expressions of PGK1 and SNHG17. *P < 0.05; **P < 0.01; ***P < 0.001; ****P < 0.0001.

**Figure S9 SNHG17 interacts with PGK1 protein in THP-1 derived TAMs.**

(A-B) Relative mRNA expression in THP-1 derived TAMs (shNC, sh1-SNHG17, sh2-SNHG17) co-cultured with PANC-1 (A) and PATU-8988 (B). (C) Western Blot showing the relative protein expressions of PGK1. (D-E) Relative mRNA expression in THP-1 derived TAMs (NC, SNHG17-OE) co-cultured with PANC-1 (D) and PATU-8988 (E). (F) Western Blot showing the relative protein expressions of PGK1. (G-H) Relative mRNA expression in THP-1 derived TAMs (NC, PGK1-OE) after co-cultured with PANC-1 (G) and PATU-8988 (H). (I) Western Blot showing the relative protein expressions of PGK1. *P < 0.05; **P < 0.01; ***P < 0.001; ****P < 0.0001.

**Figure S10 PGK1 in THP-1 derived TAMs promotes M2 polarization and PDAC proliferation.**

(A-B) The mRNA expression of M2 markers (CD206, CD163, IL6, IL10, TGFB and Arg1) and M1 markers (IL1B and CD80) in TAMs after co-culture with PANC-1 (A) and PATU-8988 (B). (C-J) Flow Cytometry analysis of CD80, CD86, CD163, CD206 expression in THP-1 derived TAMs co-cultured with PANC-1 (C-F) and PATU-8988 (G-J). (K) Colony formation images of PANC-1 and PATU-8988 co-cultured with TAMs (shNC, sh1-PGK1, sh2-PGK1). (L-M) Relative colony numbers of PANC-1 (L) and PATU-8988 (M) after co-cultured with TAMs. (N-O) CCK8 assay analysis of PANC-1 (N) and PATU-8988 (O) after co-cultured with TAMs (shNC, sh1-PGK1, sh2-PGK1). *P < 0.05; **P < 0.01; ***P < 0.001; ****P < 0.0001.

**Figure S11 PGK1 promotes the proliferation of patient derived PDAC organoids.**

(A-B) CTG assay analysis of PDO1# (A) and PDO2# (B) co-cultured with TAMs (shNC, sh1-PGK1, sh2-PGK1). (C-D) Representative pictures of PDO1#(C) and PDO2#(D) after co-cultured with TAMs (shNC, sh1-PGK1, sh2-PGK1) in day0, day5 and day10. (E-F) Relative areas of co-cultured PDO1# (E) and PDO2# (F). *P < 0.05; **P < 0.01; ***P < 0.001; ****P < 0.0001.

**Figure S12 PGK1 promotes migration, invasion, glucose uptake and LA release.**

(A) Migration and invasion of PANC-1 and PATU-8988 co-cultured with THP-1 derived TAMs (shNC, sh1-PGK1, sh2-PGK1). (B-C) Migrated and invaded cells per field of PANC-1 and PATU-8988 co-cultured with THP-1 derived TAMs. (D-E) Glucose uptake capacity of PANC-1 (D) and PATU-8988 (E) after co-cultured with TAMs. (F-G) Lactic acid concentration of PANC-1 (F) and PATU-8988 (G) after co-cultured with TAMs. *P < 0.05; **P < 0.01; ***P < 0.001; ****P < 0.0001.

**Figure S13 Binding ability of SNHG17 to PGK1 or PGK1 mutations.**

(A-B) Binding ability of SNHG17 to PGK1 or PGK1 mutated at the T281A site (A) and T378A (B) analyzed by RNA pull-down assay.

**Figure S14 SNHG17 in TAMs binds to PGK1 to promote M2 polarization and proliferation of PCs through T168A of PGK1.**

(A-D) Flow Cytometry analysis of CD80 (A), CD86 (B), CD163 (C), CD206 (D) expression in THP-1 derived TAMs (NC, PGK1 WT and PGK1 MUT) co-cultured with PATU-8988. (E) The mRNA expression of M2 markers (CD206, CD163, IL6, IL10, TGFB and Arg1) and M1 markers (IL1B and CD80) in TAMs after co-cultured with PATU-8988. (F-G) CTG assay analysis of PDO1# (F) and PDO2# (G) co-cultured with TAMs (NC, PGK1 WT and PGK1 MUT). (H-I) Relative areas of co-cultured PDO1# (H) and PDO2# (I). (J-K) Representative pictures of PDO1# (J) and PDO2# (K) after co-cultured with TAMs (NC, PGK1 WT and PGK1 MUT) in day0, day5 and day10. *P < 0.05; **P < 0.01; ***P < 0.001; ****P < 0.0001.

**Figure S15 SNHG17 binds to PGK1.**

(A) Co-IP analysis of THP-1 derived TAMs (NC and SNHG17-OE) after co-cultured with PANC-1 and PATU-8988. (B) Co-IP analysis of THP-1 derived TAMs (PGK1-WT-Flag, PGK1-T168A-Flag, PGK1-T281A-Flag and PGK1-T378A-Flag) after co-cultured with PANC-1 and PATU-8988.

**Figure S16 SNHG17 binds to PGK1 to enhance the phosphorylation of PGK1.**

(A-B) Relative mRNA expression of ERK1 and ERK2 in THP-1 derived TAMs (NC and SNHG17-OE) after co-cultured with PANC-1 (A) and PATU-8988 (B). (C-D) Relative mRNA expression of ERK1 and ERK2 in THP-1 derived TAMs (shNC, sh1-SNHG17 and sh2-SNHG17) after co-cultured with PANC-1 (C) and PATU-8988 (D). (E-F) Relative mRNA expression of ERK1 and ERK2 in THP-1 derived TAMs (shNC, sh1-PGK1, sh2-PGK1) after co-cultured with PANC-1 (E) and PATU-8988 (F). (G) WB analysis of THP-1 derived TAMs. (H) RNA pull-down analysis of THP-1 derived TAMs (NC and ERK1/2-OE). (I) Relative mRNA expression of SNHG17 in THP-1 derived TAMs (NC and ERK1/2-OE) by RIP assay. (J) Relative mRNA expression of SNHG17 in THP-1 derived TAMs (NC, sh1-ERK1/2 and sh2-ERK1/2) by RIP assay. *P < 0.05; **P < 0.01; ***P < 0.001; ****P < 0.0001.
