# Supplementary material for: SNHG17 alters anaerobic glycolysis by resetting phosphorylation modification of PGK1 to foster pro-tumor macrophage formation in pancreatic ductal adenocarcinoma
Source: J Exp Clin Cancer Res. 2023 Dec 15;42:339. doi: 10.1186/s13046-023-02890-z (PMC10722693; doi:10.1186/s13046-023-02890-z)
Supplement: Supplementary file 8 — Additional file 8: Figure S8. SNHG17 sponges miR-628-5p to release PGK1 mRNA. [file 13046_2023_2890_MOESM8_ESM.pdf]

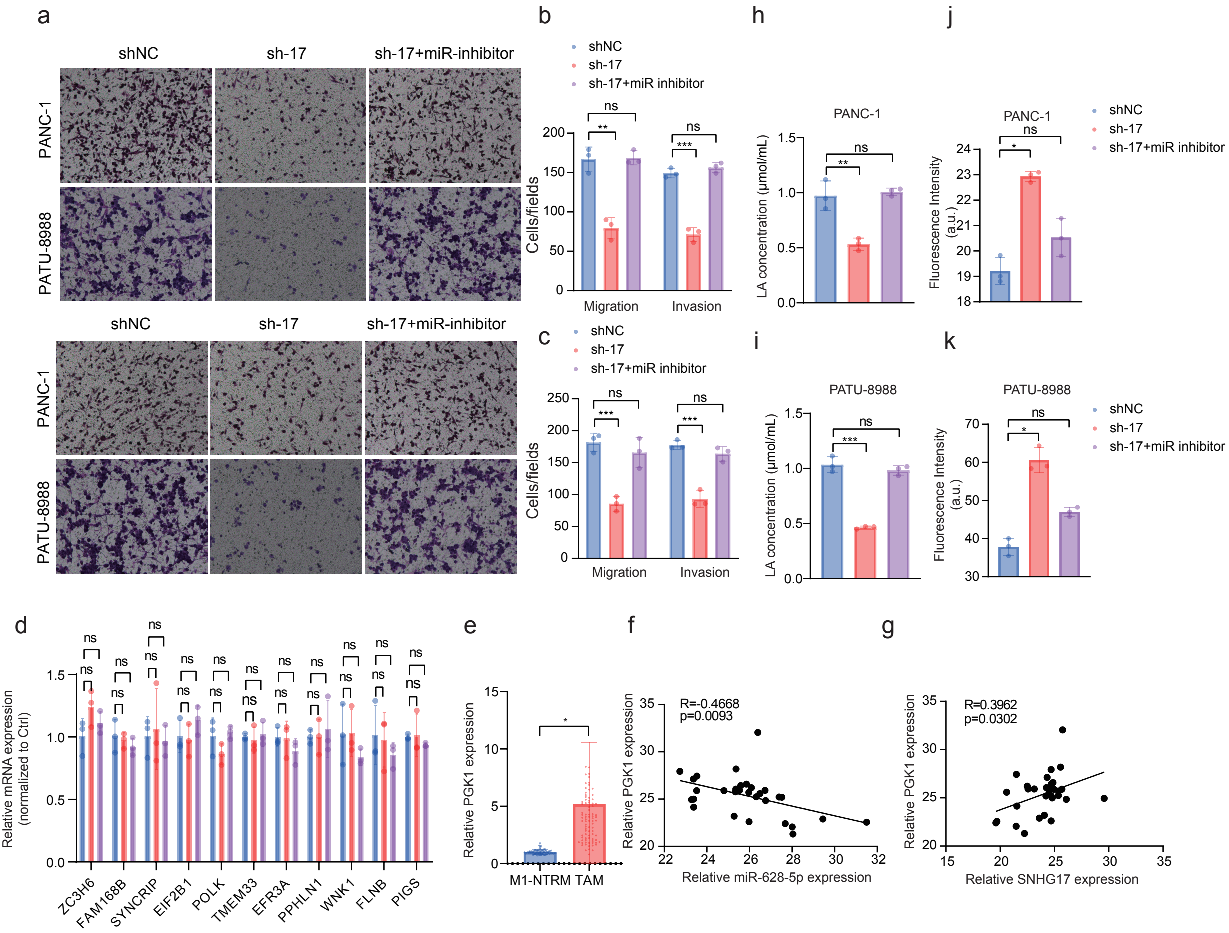

Supplementary Figure 8
